# The Swinholide Biosynthesis Gene Cluster from a Terrestrial Cyanobacterium, Nostoc sp. Strain UHCC 0450

**DOI:** 10.1128/AEM.02321-17

**Published:** 2018-01-17

**Authors:** Anu Humisto, Jouni Jokela, Liwei Liu, Matti Wahlsten, Hao Wang, Perttu Permi, João Paulo Machado, Agostinho Antunes, David P. Fewer, Kaarina Sivonen

**Affiliations:** a Department of Food and Environmental Sciences, Viikki Biocenter 1, University of Helsinki, Helsinki, Finland; b Program in Structural Biology and Biophysics, Institute of Biotechnology, University of Helsinki, Helsinki, Finland; c Department of Biological and Environmental Science, Nanoscience Center, University of Jyväskylä, Jyväskylä, Finland; d Department of Chemistry, Nanoscience Center, University of Jyväskylä, Jyväskylä, Finland; e CIIMAR/CIMAR, Interdisciplinary Centre of Marine and Environmental Research, University of Porto, Porto, Portugal; f Department of Biology, Faculty of Sciences, University of Porto, Porto, Portugal; University of Bayreuth

**Keywords:** cyanobacteria, *Nostoc*, “*Candidatus* Entotheonella”, polyketides, *trans*-AT PKS, swinholide, scytophycin, horizontal gene transfer, *Anabaena*

## Abstract

Swinholides are 42-carbon ring polyketides with a 2-fold axis of symmetry. They are potent cytotoxins that disrupt the actin cytoskeleton. Swinholides were discovered from the marine sponge *Theonella* sp. and were long suspected to be produced by symbiotic bacteria. Misakinolide, a structural variant of swinholide, was recently demonstrated to be the product of a symbiotic heterotrophic proteobacterium. Here, we report the production of swinholide A by an axenic strain of the terrestrial cyanobacterium Nostoc sp. strain UHCC 0450. We located the 85-kb *trans*-AT polyketide synthase (PKS) swinholide biosynthesis gene cluster from a draft genome of Nostoc sp. UHCC 0450. The swinholide and misakinolide biosynthesis gene clusters share an almost identical order of catalytic domains, with 85% nucleotide sequence identity, and they group together in phylogenetic analysis. Our results resolve speculation around the true producer of swinholides and demonstrate that bacteria belonging to two distantly related phyla both produce structural variants of the same natural product. In addition, we described a biosynthesis cluster from Anabaena sp. strain UHCC 0451 for the synthesis of the cytotoxic and antifungal scytophycin. All of these biosynthesis gene clusters were closely related to each other and created a group of cytotoxic macrolide compounds produced by *trans*-AT PKSs of cyanobacteria and proteobacteria.

**IMPORTANCE** Many of the drugs in use today originate from natural products. New candidate compounds for drug development are needed due to increased drug resistance. An increased knowledge of the biosynthesis of bioactive compounds can be used to aid chemical synthesis to produce novel drugs. Here, we show that a terrestrial axenic culture of Nostoc cyanobacterium produces swinholides, which have been previously found only from marine sponge or samples related to them. Swinholides are polyketides with a 2-fold axis of symmetry, and they are potent cytotoxins that disrupt the actin cytoskeleton. We describe the biosynthesis gene clusters of swinholide from Nostoc cyanobacteria, as well as the related cytotoxic and antifungal scytophycin from Anabaena cyanobacteria, and we study the evolution of their *trans*-AT polyketide synthases. Interestingly, swinholide is closely related to misakinolide produced by a symbiotic heterotrophic proteobacterium, demonstrating that bacteria belonging to two distantly related phyla and different habitats can produce similar natural products.

## INTRODUCTION

Cyanobacteria produce structurally diverse secondary metabolites with a range of potent biological activities (1–3). Many of these secondary metabolites are polyketides with clinically important bioactivities (4, 5). For instance, several antibiotic, antitumor, and antifungal polyketides, as well as potential toxins, are found from bacteria, fungi, and plants (4–6). Polyketides are synthesized on multidomain polyketide synthase (PKS) enzyme complexes that build polyketides from small monomeric constituents through chain elongation (4, 7, 8). Each PKS module adds either an acyl, malonyl, or derivative unit into the growing polyketide chain. PKSs are divided into three different classes (I to III) based on their domain architecture and function (4, 9). Type I polyketide synthases include *cis*- and *trans*-acyltransferase (*trans*-AT) PKSs. Each module of the *cis*-AT PKSs encodes a dedicated AT domain, while the *trans*-AT PKSs have discrete ATs that are used iteratively in place of the *cis*-encoded AT domains (4, 5, 10). The *trans*-AT PKSs deviate from the *cis*-AT PKS colinearity rules, and the modular architecture of these enzymes can be more varied (5). Many bacterial *trans*-AT PKSs have been described to date from sources, such as symbiotic and soil bacteria, and they often have potent bioactivities, such as toxin and antibiotic activities (5).

**FIG 1:**
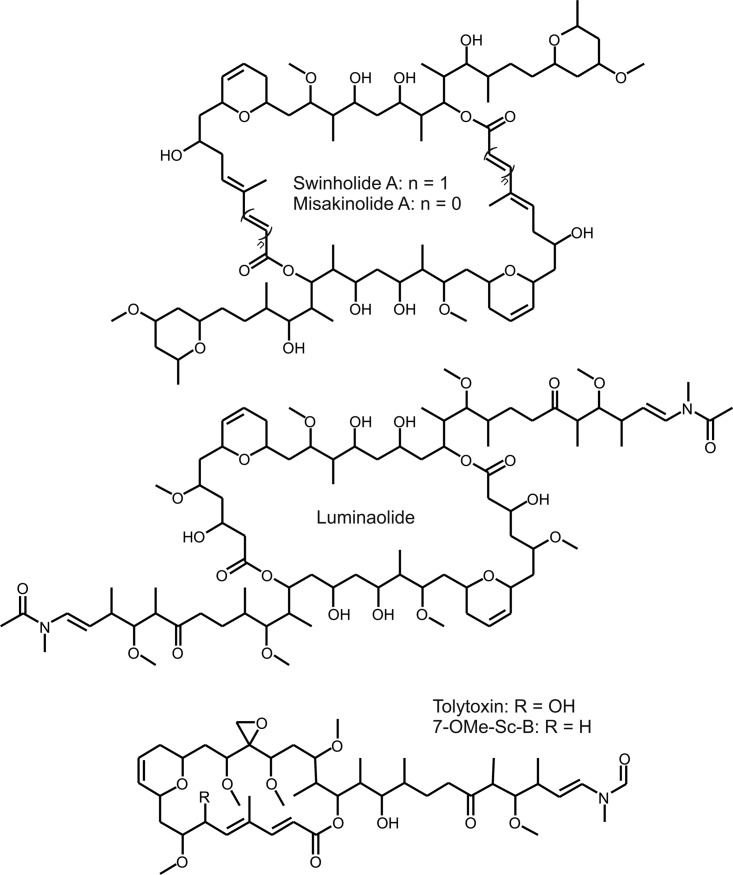
Structures of polyketide compounds swinholide, misakinolide, luminaolide, and scytophycins (Sc).

Swinholide (Fig. 1) is a polyketide with a dimeric 42-carbon ring structure exhibiting a 2-fold axis of symmetry (11, 12). This macrolide natural product has cytotoxic and antifungal activities through the disruption of the actin cytoskeleton (11, 13, 14). Swinholide was first described in 1985 (11), the structure was corrected in 1989 (12), and the stereochemistry was solved in 1990 (15). Thirteen swinholides and a few close structural analogues, such as misakinolides/bistheonellides (16, 17), ankaraholides (18), and hurghadolide A (19), have been described in the literature (Fig. 1, Fig. S1A in the supplemental material, and Table 1). Most swinholides and structural variants have been found from the marine sponge *Theonella*. Symbiotic microbes were long suspected to produce swinholides and other bioactive compounds, since the marine sponges, such as *Theonella* species, are well known for their capacity to host large amounts of symbionts (15, 20, 21).

**TABLE 1:** Reported swinholides, their exact masses, and isolation organisms

Name	Monoisotopic mass	Organism (origin)	Reference(s)
Swinholide A	1,388.9311	*Theonella swinhoei* (Okinawa, Japan)	12, 15
		*Symploca* cf. sp. (Fiji)	18
Swinholide B	1,374.9155	*Theonella swinhoei* (Okinawa, Japan)	13
Swinholide C	1,374.9155	*Theonella swinhoei* (Okinawa, Japan)	13
Swinholide D	1,374.9155	*Theonella swinhoei* (Okinawa, Japan)	20
Swinholide E	1,404.9260	*Theonella swinhoei* (Okinawa, Japan)	20
Swinholide F	1,388.9311	*Theonella swinhoei* (Okinawa, Japan)	20
Swinholide G	1,374.9155	*Theonella swinhoei* (Okinawa, Japan)	20
Swinholide H	1,416.9624	Lamellomorpha strongylata (New Zealand)	52
Swinholide I	1,404.9260	*Theonella swinhoei* (Red Sea, Egypt)	19
Swinholide J	1,404.9260	Theonella swinhoei (Solomon Islands)	53
Swinholide K	1,404.9261	Theonella swinhoei (Indonesia)	54
Isoswinholide A	1,388.9311	*Theonella swinhoei* (Okinawa, Japan)	12
Isoswinholide B	1,388.9311	Theonella swinhoei (Indonesia)	54

Swinholide A has also been reported from a marine field sample containing the cyanobacterium *Symploca* sp. (18) (Table 1). Ankaraholides, structural variants of swinholides, were also found from the cyanobacterium Geitlerinema sp. in the same study (18). Sponges host a range of bacteria, including symbiotic cyanobacteria, raising questions about the producer of swinholides (21, 22). However, the production of misakinolide, a close structural variant of swinholide, was recently attributed to the *Theonella* symbiont bacterium “Candidatus Entotheonella” through the discovery of a *trans*-AT PKS biosynthesis gene cluster (23). The true origin of the swinholides in the field samples remains unclear. Here, we describe a swinholide biosynthesis gene cluster and report swinholide production by a pure culture of the terrestrial cyanobacterium Nostoc sp. strain UHCC 0450. Our results demonstrate that two distantly related bacteria from different bacterial phyla produce the same toxin.

## RESULTS

### Identification of swinholide.

**FIG 2:**
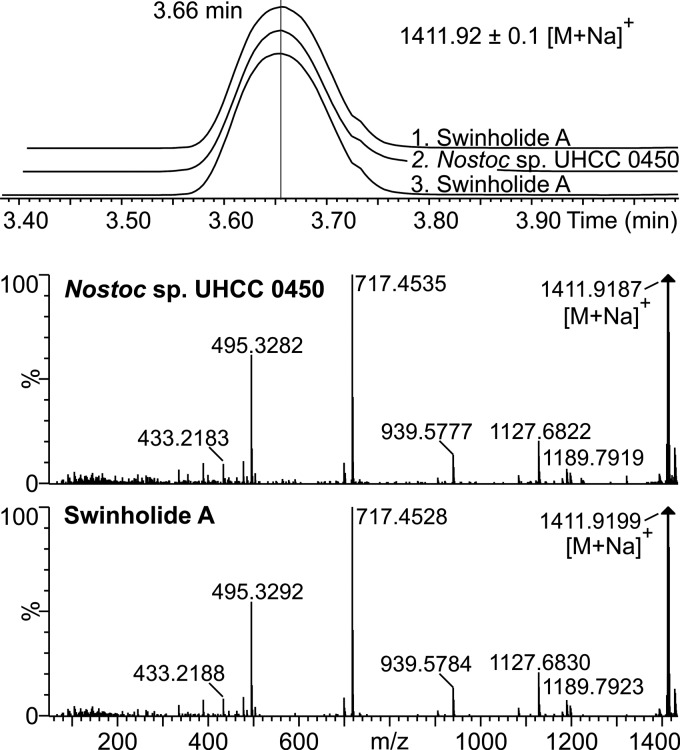
Elution (duplicate injections, *t*_R_ 3.66 min) and MS^E^ fragmentation of commercial swinholide A and the compound from Nostoc sp. UHCC 0450.

We detected antifungal activity against Aspergillus flavus from methanol extracts of Nostoc sp. UHCC 0450 (previously N107.3) and Anabaena sp. strain UHCC 0451 (previously HAN 21/1) in plate diffusion assays (24). Bioactivity-guided fractionation was used to identify candidate antifungal compounds from the extracts of these two strains. The antifungal compound from Anabaena sp. UHCC 0451 was identified as 7-OMe-scytophycin-B (24). Surprisingly, the antifungal compound from a pure culture of Nostoc sp. UHCC 0450 was identified as a swinholide. Ultraperformance liquid chromatography–high-resolution mass spectrometry (UPLC-HRMS) results showed that the methanol extract of Nostoc sp. UHCC 0450 contained a compound whose retention time, protonated mass (*m/z* 1,389.9), and fragmentation were practically identical to those of commercial swinholide A (Fig. 2 and S1B). Four swinholide isoforms, swinholide A, swinholide F, and isoswinholides A and B, have identical masses and elemental compositions, making it impossible to assign a chemical structure by high-resolution liquid chromatography-mass spectrometry (HR-LCMS) alone (Table 1). Therefore, we carried out nuclear magnetic resonance (NMR) analysis to determine conclusively the chemical structure of the swinholide variant produced by Nostoc sp. UHCC 0450. NMR analysis demonstrated that the swinholide variant was swinholide A, which is the same variant reported from the field sample containing the cyanobacterium *Symploca* sp. and the marine sponge *Theonella* (Fig. S2 and Table S1). The other swinholide variants with an identical elemental composition were ruled out by NMR analysis (Table S1). Minor swinholide variants recognized by their similar product ion spectra of the sodiated molecules with swinholide A constituted less than 20% of the total amount of swinholides produced by Nostoc sp. UHCC 0450 (Fig. S3 and Table S2). The minor swinholide variants produced by Nostoc sp. UHCC 0450 vary most probably in the methyl/methoxy and hydroxyl/epoxy groups (Fig. S4 and references in Table 1). The mass accuracy was not high enough to unequivocally determine the elemental composition of the variants found, but at least swinholide variant 3 is new, based on the previously reported masses alone (Tables 1 and S2). Monomeric seco acid of swinholide A, which was identified from samples of the Okinawan marine sponge *Theonella* sp. (20), was not detected from Nostoc sp. UHCC 0450.

### Swinholide biosynthesis gene cluster.

We obtained a draft genome sequence of axenic Nostoc sp. UHCC 0450 in order to identify the swinholide biosynthesis gene cluster. The draft genome was 7.32 Mb in size, with 105 scaffolds. The swinholide biosynthesis gene cluster (*swi*) was located on a single scaffold by BLASTp searches against the recently published misakinolide biosynthesis gene cluster genes due to the close structural resemblance of these molecules (23). The three gaps in the swinholide biosynthesis gene cluster were closed by PCR and Sanger sequencing. One of the three gaps was located inside *swiD*, and the sequence data indicated that there were four 21-bp identical repeats encoding the GTGDWGL motif.

**FIG 3:**
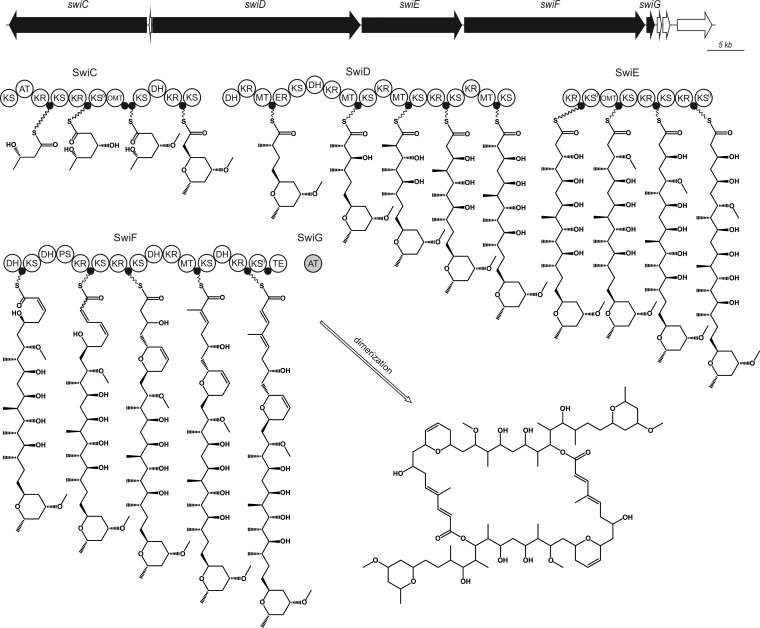
The organization of the putative 85-kb swinholide (*swi*) biosynthetic gene cluster and the proposed biosynthesis pathway of swinholide in Nostoc sp. UHCC 0450. AT, acyltransferase; DH, dehydratase; ER, enoyl reductase; KR, ketoreductase; KS, ketosynthase, KS^0^, nonelongating KS; MT, methyltransferase; OMT, *O*-methyltransferase; PS, pyransynthase; TE, thioesterase; ●, acyl carrier protein.

**FIG 4:**
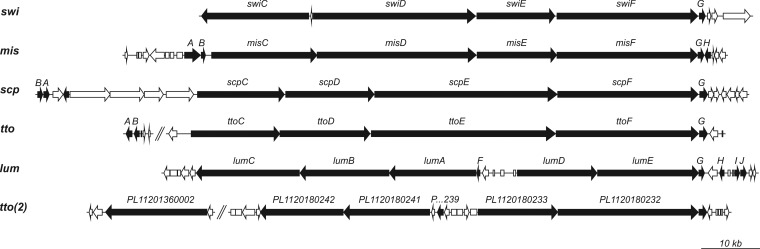
Comparison of the organization of the *trans*-AT PKS genes used in phylogenetic analysis. Gene clusters of swinholide (*swi*), misakinolide (*mis*), scytophycin (*scp*), tolytoxin (*tto1* and *tto2*), and luminaolide (*lum*) are presented.

**FIG 5:**
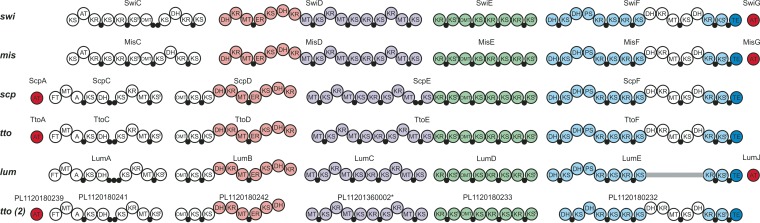
Domains of the six related biosynthesis gene clusters. Swinholide biosynthesis gene cluster (*swi*) found from Nostoc sp. UHCC 0450, misakinolide (*mis*) from “Candidatus Entotheonella” sp. TSWA-1, scytophycin (*scp*) from Anabaena sp. UHCC 0451, tolytoxin (*tto*) from Scytonema sp. PCC 10023, luminaolide (*lum*) from Planktothrix paucivesiculata PCC 9631, and second tolytoxin (*tto2*) from *Planktothrix* sp. PCC 11201 (*, gene for PL11201360002 sequence is located in a different contig). AT, acyltransferase; DH, dehydratase; ER, enoyl reductase; KR, ketoreductase; KS, ketosynthase, KS^0^, nonelongating KS; MT, methyltransferase; OMT, *O*-methyltransferase; PS, pyran synthase; TE, thioesterase; ●, acyl carrier protein.

The 85-kb swinholide biosynthesis gene cluster encodes five PKS proteins (SwiC to SwiG), including a standalone AT enzyme (SwiG), which is a hallmark of *trans*-AT PKSs (Fig. 3). The swinholide biosynthesis gene cluster thus encodes a *tran*s-acyltransferase PKS and lacks integrated AT domains similar to the misakinolide (*mis*), tolytoxin (*tto*), luminaolide (*lum*), nosperin (*nsp*), and phormidolide (*phm*) clusters (23, 25, 26). It is especially similar to the recently identified misakinolide biosynthesis gene cluster, as well as tolytoxin and luminaolide clusters (Fig. 4 and 5 and Table S3A) (23). The order of the genes in swinholide and misakinolide biosynthesis gene clusters differs, but both consist of four large genes encoding PKS enzymes, followed by a gene encoding the acyltransferase protein (Fig. 4). In the misakinolide biosynthesis gene cluster, all the genes are orientated in the same direction, whereas in the swinholide biosynthesis gene cluster, the first gene, *swiC*, is on the reverse strand and the other four genes are following in the forward direction. However, the proteins encoded by the swinholide and misakinolide biosynthesis gene clusters contain a very similar set of catalytic domains, which was expected, as these two polyketides differ only by two –HC=CH– units (Fig. 1 and 5).

The swinholide biosynthesis enzymes have an unusual domain order, split modules, and nonelongating domains, which are typical characteristics of a *trans*-AT PKS. There are four nonelongating ketosynthases (KS^0^s) in the swinholide cluster which do not contribute to the lengthening of the polyketide chain. Three of these KS^0^s are combined with modification enzymes, and the last one is in the terminal part of SwiF. We observed only a minor difference in the organization of catalytic domains in the enzymes encoded by the misakinolide and swinholide biosynthesis gene clusters. There were two acyl carrier proteins (ACPs) in the middle of the SwiC protein, instead of a single ACP as in the corresponding MisC protein (Fig. 5).

Both swinholide and misakinolide have two different ring structures in their monomeric structures. The second and third dehydratases (DHs) in the SwiF protein were located side by side (Fig. 5). The same two dehydratase-like domains were also found in the misakinolide cluster, where the third dehydratase was identified in detail as pyran synthase (23). Pyran synthase (PS) creates the dihydropyran ring in the structure (5). By comparing sequences, we could see that the third DH in SwiF resembled the pyran synthase of misakinolide. Both of these sequences lacked the known deletion of pyran synthases in one active-site motif (HxxxGxxxxP) (5, 27), and therefore, the third dehydratase of SwiF is here also named pyran synthase (Fig. 3 and 5). The other ring formation in misakinolide was hypothesized to be catalyzed by DH in MisC or with the help of accessory enzymes, such as the putative phosphoenolpyruvate synthase MisA (23). However, none of the putative open reading frames (ORFs) in the immediate vicinity of swinholide biosynthesis gene cluster were similar to the gene encoding the MisA protein. However, MisC and SwiC encode similar aberrant DHs but lack a PS domain as in the dihydropyran ring formation (Fig. 5). The DH domains from MisC and SwiC lack the glycine in the HxxxGxxxxP motif. This might indicate that an aberrant DH domain plays a significant role in ring formation. It was not possible to differentiate *C*-methyltransferase and *O*-methyltransferase domains based on sequence, and they were predicted using the chemical structure of swinholide (Fig. 3).

The sequence identities of the core genes of the swinholide and misakinolide biosynthesis gene clusters varied from 73 to 85% despite the pronounced structural similarities between the swinholides and misakinolides (Table S3A). In addition, the scytophycin, tolytoxin, and luminaolide biosynthesis cluster genes also shared a high sequence identity to misakinolide and swinholide genes (Table S4). However, SwiC and MisC proteins differ substantially from counterparts of the other mentioned biosynthesis gene clusters, which is explained by their difference in chemical structures (Fig. 1 and 5).

### Scytophycin biosynthesis gene cluster.

We obtained a draft genome sequence also from Anabaena sp. UHCC 0451 in order to identify the scytophycin biosynthesis gene cluster and to increase the amount of *trans*-AT biosynthesis gene clusters described. The assembled draft genome was 5.74 Mb, with 77 scaffolds. The scytophycin biosynthesis gene cluster could be identified using the newly discovered tolytoxin (*tto*) gene cluster from Scytonema sp. strain PCC 10023 (23). Both of these biosynthesis gene clusters encode *trans*-AT PKSs, like the swinholide cluster. The scytophycin biosynthesis gene cluster (*scp*, 86 kb) from Anabaena sp. UHCC 0451 is almost identical to tolytoxin biosynthesis gene cluster (*tto*) from Scytonema sp. PCC 10023 (Fig. 4) (23). The scytophycin biosynthesis gene cluster is constructed of four main genes (*scpC* to *spcF*), in addition to an AT-coding gene (*scpA*). The order of these genes is identical to that of the tolytoxin genes. The *scp* genes had 76 to 81% similarity (identity) to tolytoxin genes on BLASTp searches (Table S3B). The domains in the *tto* and *scp* genes were the same, apart from *scpE* having an extra acyl carrier protein in the middle of the gene, a tandem-ACP. In addition, the surroundings of the main biosynthesis genes in *tto* and *scp* resembled each other. Similar matches, for instance, small additional genes *ttoB* (methyltransferase) and *ttoG* (cytochrome P450), were found from the Anabaena sp. UHCC 0451 sequence (*scpB* and *scpG*).

### Phylogenetic analysis of the swinholide, misakinolide, scytophycin, tolytoxin, and luminaolide biosynthesis gene clusters.

**FIG 6:**
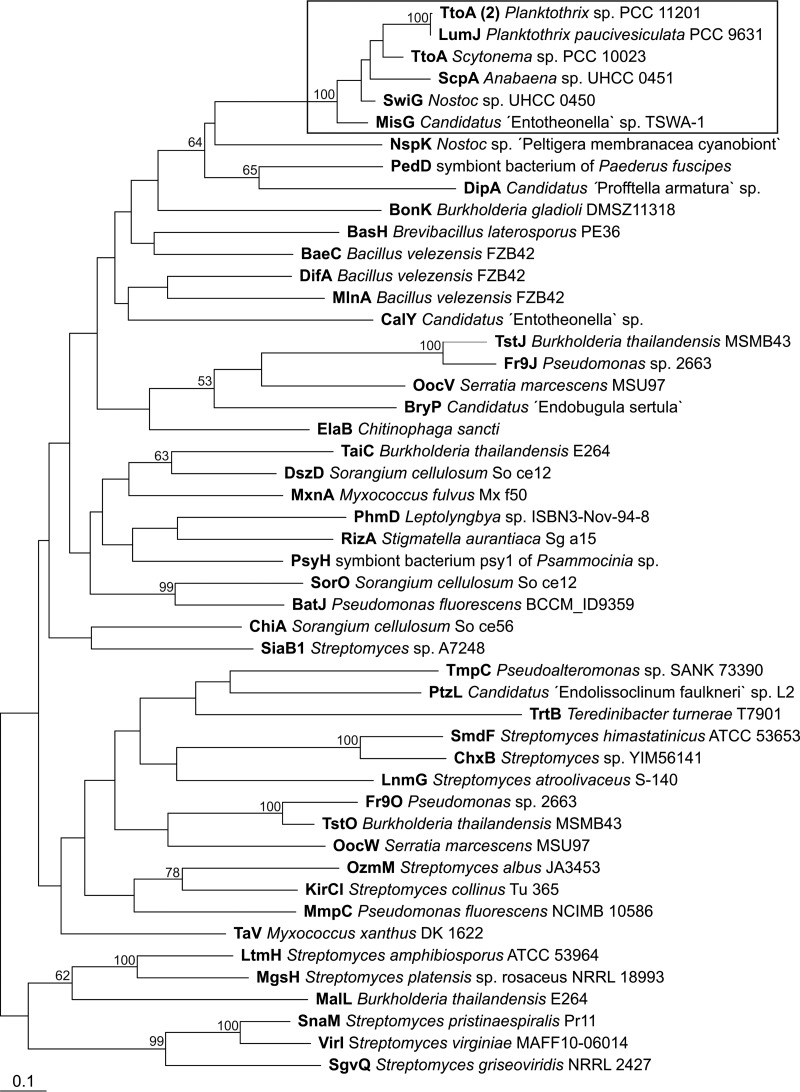
A maximum likelihood tree based on the acyltransferase (AT) proteins of different *trans*-AT PKS biosynthesis gene clusters. Swinholide-type compounds are clustered together (box). Branch lengths are proportional to sequence change, and bootstrap values above 50% are given at the node. The tree was rooted with *cis*-AT from enacyloxin gene cluster of Burkholderia ambifaria AMMD (accession no. ABI91466.1). Information about the produced compounds and the accession numbers of the proteins are provided in Table S6 in the supplemental material.

**FIG 7:**
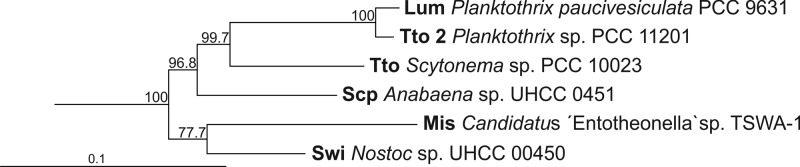
A maximum likelihood tree showing the relationship between misakinolide and swinholide biosynthesis gene clusters. The maximum likelihood tree was constructed of the ketosynthases shared by all six biosynthesis gene clusters, KS6 to KS19 (not including KS18, due to the lack of its presence in all clusters). Branch lengths are proportional to sequence change, and bootstrap values above 50% are given at the node. Two outgroup sequences (not shown) were constructed manually by using closest BLAST hits to each ketosynthase domain of the misakinolide biosynthesis enzymes.

The closely related swinholide, misakinolide, scytophycin, tolytoxin, and luminaolide biosynthesis gene clusters were compared in order to gain insights into the origins of the swinholides and misakinolides. A maximum likelihood phylogenetic tree of *trans*-encoded AT proteins showed that all six biosynthesis gene clusters were close together and formed their own well-supported group (Fig. 6). These gene clusters were clearly separated from other *trans*-ATs shown in the tree, but this analysis did not resolve the relationship between swinholide and misakinolide gene clusters, as the bootstrap values were under 50% (Fig. 6). We constructed two rooted maximum likelihood trees based on 13 KS domains common to all six biosynthesis gene clusters (Fig. 7 and S4). All ketosynthase domains were very similar, and domains from different biosynthesis gene clusters with the same number clustered together (Fig. S4). This allowed us to prepare a concatenated alignment of the 13 KS domains. We obtained an alignment comprising 5,000 positions that was used to construct the second maximum likelihood tree (Fig. 7). The scytophycin, tolytoxin, and luminaolide biosynthesis gene clusters grouped together based on ketosynthase domains, and the misakinolide and swinholide biosynthesis gene clusters formed their own group but with a lower bootstrap value (Fig. 7).

### Horizontal gene transfer and codon bias.

The χ^2^ test obtained a *P* value of <2.2E^−16^ for the raw comparison of the swinholide cluster with the genome of Nostoc sp. UHCC 0450 and, after normalization, a *P* value of 6.0E^−09^. This suggests that the codon usage in PKS is significantly different from the codon usage observed in the genome of Nostoc sp. UHCC 0450. However, a donor species could not be assigned, since the BLAST-based analysis implemented in HGTector failed to trace a horizontal gene transfer (HGT) event in any of the swinholide modules (Table S5). Given the structural similarity between the swinholide and misakinolide, a codon usage analysis was performed between the “Candidatus Entotheonella” sp. strain TSWA-1 genome and the swinholide cluster. The χ^2^ test using the raw (*P* < 2.2E^−16^) and the normalized (*P* < 2.2E^−16^) comparisons indicates that the swinholide gene cluster is also dissimilar from the “Candidatus Entotheonella” sp. TSWA-1 codon usage. Therefore, the results imply that the structural and activity similarities observed from swinholide and misakinolide, if explained by HGT, originate from a third source and not from direct HGT between these two species. Concordantly the codon usage analysis from the misakinolide gene cluster showed differences from the “Candidatus Entotheonella” sp. TSWA-1 using either the raw or normalized comparison. HGTector analysis reported evidence for a HGT event from Oscillatoriales for the *misE* and *misG* genes (Table S5). This suggests that this taxon is the best candidate donor group given the similarities between the swinholide and misakinolide. Nevertheless, the HGTector did not provide evidence that Oscillatoriales is the source of any of the modules of swinholide.

The results from HGTector indicate the occurrence of HGT events that affect the scytophycin genes: *scpC* from an Actinomycetales bacterium, *scpD* from a Thiotrichales bacterium, and *scpF* from a Burkholderiales bacterium (Table S5). In luminaolide, an HGT event was detected from a Flavobacteriales bacterium for *lumJ* module (Table S5). An HGT event, from the module *ttoD* from a Thiotrichales donor, was detected for tolytoxin 1 (Table S5). In contrast, HGT events for tolytoxin 2 were reported from a Burkholderiales donor for the module *tto2D* and from a Sphingomonadales donor for the module *tto2F* (Table S5). Overall, the results show evidence of HGT events in several PKS modules and therefore highlight important events in the evolution of these gene clusters.

## DISCUSSION

Here, we report the discovery of swinholide A from a pure (axenic) culture of the lichen cyanobacterium Nostoc sp. UHCC 0450. Almost all swinholides found to date have been extracted from marine sponge *Theonella* sp. samples (Table S1), including the first report of swinholide A from *Theonella* sp. (11, 12). However, there has been much speculation surrounding the nature of the swinholide producer. Symbiotic organisms were long suspected to be the true producers of swinholide, and swinholide production has been attributed to cyanobacterial symbionts as well as filamentous heterotrophic bacterial symbionts of sponges (15, 18, 21). A heterotrophic bacterial origin has been suggested, since swinholide was found from a unicellular bacterial fraction of the sponge Theonella swinhoei (21). Unequivocal evidence for the production of swinholide analog by symbiotic bacteria came with the recent discovery of a biosynthesis gene cluster of a close structural variant, misakinolide A, from the symbiotic bacterium “Candidatus Entotheonella serta” TSWA-1, isolated from Theonella swinhoei (23). Here, we show that cyanobacteria can also produce swinholide. Cyanobacteria were seen as strong candidates for producing swinholide, since swinholide was isolated from a field sample of the cyanobacterium *Symploca* sp. and ankaraholides A and B from the cyanobacterium Geitlerinema sp. (18). It has been also suggested that multiple classes of bacteria could produce swinholides due to gene transfer events (18). Our results confirm this hypothesis, but substantial differences between the two gene clusters identified here together with an analysis of HGT suggest that this is more complex than a simple recent transfer of the gene cluster from one phylum to another (Table S5).

The swinholide-producing strain Nostoc sp. UHCC 0450 was isolated from lichen. Nostoc cyanobacteria are known from freshwater and soil and are commonly found in lichen symbioses (28). Lichen-symbiotic Nostoc spp. have been found to produce important compounds, such as hepatotoxic microcystins and clinically important cryptophycins (28, 29). In addition, another *trans*-AT PKS product, nosperin, was found from symbiotic Nostoc cyanobacteria in the lichen Peltigera membranacea (25). This suggests that these types of biosynthesis gene clusters or compounds are especially common among symbiotic strains. However, phylogenetic differences between free-living and symbiotic Nostoc strains have not been detected (30). Symbiosis provides an environment where different species come into close contact and may promote gene transfer, regardless of the roles the bioactive compounds play in symbiosis.

The biosynthesis gene cluster of swinholide from Nostoc sp. UHCC 0450 is increasing the group of *trans*-AT PKS clusters that have been identified from cyanobacteria in addition to nosperin, tolytoxin, luminaolide, and phormidolide clusters from Nostoc spp., Scytonema spp., *Planktothrix* paucivesiculata, and Leptolyngbya spp., respectively (23, 25, 26). The swinholide biosynthesis gene cluster had an organization of catalytic domains that was almost identical to that of the previously described misakinolide cluster (23). The chemical structures of swinholide and misakinolide differ only by a single double bond in each monomer structure (Fig. 1). Therefore, module skipping during misakinolide biosynthesis was proposed to explain the presence of an additional module in the misakinolide biosynthesis gene cluster (23), which is consistent with our findings. Consequently, all modules are needed in the production of swinholide. The swinholide cluster described here was similar to the cyanobacterial scytophycin, tolytoxin, and luminaolide biosynthesis gene clusters, which also produce macrolide compounds (23, 31). The swinholide biosynthesis gene cluster had several typical characters of a *trans*-AT PKS gene cluster, including unusual domain orders, split modules, and nonelongating modules (5). In addition to nonelongating ketosynthases, other modification enzymes were present in the swinholide cluster, such as pyran synthase and an aberrant dehydratase involved in ring constructions. Tandem-ACPs were identified in the SwiC protein (Fig. 3 and 5). The TtoC protein in tolytoxin biosynthesis gene cluster had also two ACPs following each other (23). Tandem-ACP structures are indicated to overcome rate-limiting steps in production, and these double or triple structures have been identified in many *trans*-AT PKSs (5, 32).

We also described here the biosynthesis gene cluster for scytophycin from the cyanobacterium Anabaena sp. UHCC 00451. Scytophycins were originally found from the cyanobacterium Scytonema sp. (33), and in total, 34 scytophycin variants have been reported in the literature (24). Structural analogs of scytophycins include lobophorolide and sphinxolide (34, 35). The scytophycin biosynthesis gene cluster was almost identical to the recently revealed biosynthesis gene cluster of Scytonema sp. PCC 10023, which was connected to tolytoxin production (23). Tolytoxin, also known as 6-hydroxy-7-OMe-scytophycin B, belongs to the scytophycin compounds (36). The main scytophycin variant in Anabaena sp. UHCC 00451 was 7-OMe-scytophycin-B (24). In addition, the Scytonema sp. PCC 10023 and Anabaena sp. UHCC 00451 strains produced other variants of scytophycins (23, 24). Thereby, both of these biosynthesis clusters can be considered to produce scytophycins.

Natural products are typically specific for an organism or a set of closely related organisms. However, there are some examples of natural products that are shared between distantly related organisms. Saxitoxins, the paralytic shellfish poisons, are shared between cyanobacteria and dinoflagellates (37). Geosmin, an odorous terpenoid, is produced by several eukaryotic and prokaryotic organisms, including cyanobacteria (38). Here, we report evidence for the production of a polyketide by distantly related bacteria belonging to different phyla. The *trans*-AT PKSs are widespread in bacteria, and it is indicated that they evolved independently from *cis*-AT PKSs (5, 39). The phylogenetic analysis, while based on a limited number of biosynthesis gene clusters, was not able to reveal the origin of the gene clusters. The uncultured bacteria of “Candidatus Entotheonella” have shown evidence of the production of a large variety of compounds found from their sponge hosts (40). Chemical variations were detected even among the “Candidatus Entotheonella” bacteria described from the same sponge individual (40). It is long suspected that *trans*-AT PKSs are spread through horizontal gene transfer (39), and here, we present evidence for the horizontal transfer of such a biosynthesis gene cluster.

In summary, our findings illustrate a remarkable conservation in the organization of the swinholide and misakinolide biosynthesis gene clusters spanning two distantly related bacterial phyla and resolve confusion about the true producer of swinholides. In addition, we described yet another biosynthesis cluster for scytophycin/tolytoxin compounds. All of these biosynthesis gene clusters were closely related to each other and created a group of cytotoxic macrolide compounds produced by *trans*-AT PKSs of cyanobacteria and proteobacteria. The terrestrial origin of Nostoc sp. UHCC 0450 compared to previous marine findings highlights that closely related compounds can be found from distant places and organisms.

## MATERIALS AND METHODS

### Strains and cultivation.

Nostoc sp. UHCC 0450 (previously named Nostoc sp. 107.3) was isolated from a Finnish lichen sample and purified until an axenic culture was obtained. Anabaena sp. UHCC 0451 (previously named Anabaena sp. HAN21/1) was isolated from a gastropod collected from the Finnish Baltic Sea coast (24) and was purified to axenic culture. The strains were maintained in 40 ml of Z8X liquid culture medium. The strains were then grown in 3-liter batches of Z8X medium for 30 days, collected by centrifugation for 10 min at 8,000 × *g*, and freeze-dried, in order to obtain enough biomass for NMR analysis and DNA extraction. Cultivations were made at a photo irradiance of 8 to 20 μmol · m^−2^ · s^−1^ at 20 to 25°C.

### Extraction and LC-MS.

Fifty milligrams of freeze-dried cells was extracted with 1 ml of MeOH by homogenizing with FastPrep (MP Biomedicals) at 6.5 ms^−1^ for 60 s. This suspension was centrifuged at 20,000 × *g* for 5 min, and the supernatant was used for liquid chromatography-mass spectrometry (LC-MS) analyses. Commercial swinholide A, isolated from Theonella swinhoei, was used as a reference compound (Enzo Life Sciences, Inc.).

Methanol extracts were first analyzed with high-performance liquid chromatography–electrospray ionization–low-resolution ion trap mass spectrometry (LC-ESI-ITMS; Agilent 1100 series LC/MSD Ion Trap XCT Plus; Agilent Technologies, Palo Alto, CA, USA). A 10-μl sample was injected to Luna C_8_ (2) column (2 by 150 mm, 5 μm; Phenomenex, Torrance, CA, USA), which was eluted from 5% isopropanol (+ 0.1% HCOOH, solvent B) to 100% of B in 35 min at 40°C, with a flow rate of 0.15 ml · min^−1^. Solvent A was 0.1% HCOOH. Mass spectral data were accumulated in the Ultra Scan positive electrospray ionization mode (*m/z* 26,000 · s^−1^), with a scan range of *m/z* 50 to 2,200 and by averaging four spectra.

High-resolution UPLC-quadrupole time of flight (UPLC-QTOF) analyses were performed with the Acquity I-Class UPLC Synapt G2-Si HDMS (Waters Corp., Milford, MA, USA) system. A 1-μl sample was injected to Cortecs UPLC C_18_^+^ column (2.1 by 50 mm, 1.6 μm; Waters Corp.), which was eluted at 40°C, with a flow rate of 0.3 ml · min^−1^, from 50% acetonitrile-isopropanol (1:1, + 0.1% HCOOH, solvent B) to 80% of B in 6 min, to 100% of B in 0.01 min, held for 1.99 min, back to 50% of B in 0.5 min, and finally held for 2.5 min before next run. Solvent A was 0.1% HCOOH. QTOF was calibrated with sodium formate, giving a calibrated mass range from *m/z* 91.000 to 1,921.390. Leucine enkephalin was used as a lock mass reference compound. Mass spectral data were accumulated in positive electrospray ionization resolution mode at a scan range of *m/z* 50 to 2,000. Product ion spectra were produced with the MS^E^ technique, which is similar to tandem MS (MS/MS) but without precursor selection.

### Swinholide A purification.

One gram of freeze-dried cells was extracted with 2 × 45 ml of methanol for 1 min using a SilentCrusher M homogenizer (Heidolph Instruments GmbH & Co., Germany). The sample was centrifuged at 8,000 × *g* for 10 min, and the supernatant was removed with a stream of air. The dry residue was suspended in 10 ml of 70% aqueous acetonitrile with 0.1% HCOOH. The solution was centrifuged at 20,000 × *g* for 5 min, and the supernatant was injected into a Luna C_8_ column (150 by 10 mm, 5 μm; Phenomenex, Torrance, CA, USA) in batches of 1 ml. The column was eluted isocratically with 70% aqueous acetonitrile (ACN) with 0.1% HCOOH at speed of 3.5 ml · min^−1^ at 30°C. The column was washed with 85% aqueous ACN between injections. The pooled fractions were injected into a Zorbax SB-C column (250 by 4.6 mm, 3.5 μm; Agilent Technologies, USA) in batches of 100 μl because of insufficient purity of the collected material. The column was eluted with 70% aqueous ACN with 0.1% HCOOH at a speed of 1 ml · min^−1^ at 30°C. The collected fractions were pooled and dried, and the final yield of swinholide A was 0.9 mg.

### NMR spectroscopy.

Two samples were prepared and dissolved in dimethyl sulfoxide-d_6_ (DMSO-d_6_) and CD_3_OD for NMR data collection and structural characterization of swinholide A. All NMR spectra were measured using a Varian INOVA 800 MHz NMR spectrometer, equipped with cryogenically cooled ^1^H, ^13^C, ^15^N triple resonance probe head and *z*-axis pulsed-field gradient unit. All spectra were measured at 25°C. A one-dimensional ^1^H spectrum as well as two-dimensional ^1^H, ^13^C heteronuclear single quantum coherence (HSQC) and ^1^H, ^13^C heteronuclear multiple bond correlation (HMBC) spectra were collected for resonance identification and assignment. For data collection in DMSO-d_6_ (CD_3_OD), ^1^H spectrum was accumulated with 8 (16) transients per free induction decay (FID) using 24,038 (24,038) complex points, corresponding to acquisition time of 2 (2) seconds. ^1^H, ^13^C HSQC spectra were collected with 180 (512) increments at *t*_1_ and 426 (768) complex points at *t*_2_, corresponding to acquisition times of 6 (21) ms and 85.2 (128) ms in ^13^C and ^1^H dimensions, respectively. For signal accumulation, 64 (32) transients per FID were used. For ^1^H, ^13^C HMBC data, 512 (512) *t*_1_ increments and 1,700 (2,048) complex points at *t*_2_ were collected, using 72 (256) transients per FID. This translated to acquisition times of 7.1 (7.1) ms and 340 (341.3) ms in ^13^C and ^1^H dimensions, respectively. The value for the low-pass filter in the ^1^H, ^13^C HMBC experiment was set to 8 Hz.

### DNA extraction.

The genomic DNA of Nostoc sp. UHCC 0450 was extracted using bead beating combined with the cetyltrimethylammonium bromide (CTAB) method (41). Approximately 500 ml of cultured cyanobacterial cells was harvested by centrifugation at 8,000 × *g* for 5 min due to large amounts of polysaccharides produced by Nostoc sp. UHCC 0450. The cells were washed twice with washing buffer (50 mM Tris-HCl, 100 mM EDTA, 100 mM NaCl), and the pellets were divided into Eppendorf tubes. The tubes were kept at −80°C overnight. Two different-size glass beads (acid washed, 425 to 600 μm and 710 to 1,180 μm; Sigma-Aldrich, St. Louis, MO, USA) and 800 μl of lysis buffer (100 mM Tris-HCl [pH 8], 1.5% SDS, 10 mM EDTA, 1% deoxycholate, 1% Igepal-CA630, 5 mM thiourea, 10 mM dithiothreitol) were added to the tubes. The cells were disrupted mechanically with a FastPrep-24 homogenizer (MP Biomedicals, Irvine, CA, USA) at 5 m · s^−1^ for 30 s. The tubes were placed on ice for 5 min and centrifuged for 1 min at 15,000 × *g*. The supernatant was moved to 10-ml tubes. The mechanical disruption was conducted again for the pellets with 400 μl of lysis buffer. Two hundred twenty-five microliters of 5 M NaCl and 170 μl of 10% CTAB in 0.7 M NaCl were added into each 500 ml of sample, as previously described (41). The suspensions were incubated for 20 min in 65°C. An equal amount of chloroform was added after incubation, and the tubes were mixed and centrifuged for 7 min at 10,000 × *g*. The CTAB treatment was repeated again, and after the second chloroform treatment, an equal amount of phenol-chloroform-isoamyl alcohol was added. The tubes were mixed and centrifuged for 7 min at 10,000 × *g*. The DNA was precipitated with isopropanol, and the pellet was washed with 70% ethanol. The extracted DNA was diluted into Tris-EDTA buffer, and it was further purified with RNase treatment. The genomic DNA of Anabaena sp. UHCC 0450 was isolated according to a method described earlier (42–44).

### Genome assembly and gene cluster analysis.

The purity of DNA extracts was checked using a NanoDrop 1000 spectrophotometer (Thermo Scientific) to measure the concentration and an Agilent TapeStation (Agilent Technologies) to assess the quality. High-molecular-weight DNA was subjected to library (Illumina TruSeq PCR-free 350 bp) construction and sequenced by the Illumina HiSeq 2500 platform, with a paired-end 100-cycle run (Macrogen). The raw data were first checked by Spades (version 3.7.1) for error correction and then assembled using Newbler (version 3.0). The results of the assembly were examined in Artemis (version 16.0.0; Sanger Institute), and gene clusters were identified using BLASTp searches of the National Center for Biotechnology Information (NCBI) and InterPro scans (45). Sequencing gaps in the gene cluster were closed by Sanger sequencing (Institute for Molecular Medicine Finland [FIMM]).

The identities of unusual domains in the swinholide (*swi*) biosynthesis cluster were confirmed by comparing these to corresponding normal domains from swinholide and misakinolide biosynthesis gene clusters with ClustalW multiple alignment in BioEdit (version 7.0.5.3) (46). For instance, nonelongating ketosynthases (KS^0^s) were confirmed with the observation of a conserved HGTGT motive which KS^0^s lack (5). Aberrant DH and PS domains were confirmed similarly. Distance matrices of nucleotide and amino acid sequence identities were prepared with the PROTDIST and DNADIST programs of the PHYLIP (version 3.695) package (47).

### Phylogenetic analysis.

Sequences were aligned using ClustalW and manually improved and trimmed using BioEdit (version 7.0.5.3) (46). Phylogenetic trees were constructed with PHYLIP (version 3.695) (47). Rooted maximum likelihood trees were constructed using PROML with Jones-Taylor-Thornton distances, global arrangements, and randomized input order. One thousand bootstrap replicates were obtained using neighbor-joining analysis with the SEQBOOT, PROTDIST, NEIGHBOR, and CONSENSE packages.

### Codon usage analysis and HGT.

To detect the presence of HGT, we used two different approaches previously suggested to be clear-cut and reliable (48, 49). First, we relied on a phylogeny-informed BLAST-based analysis implemented in HGTector version 0.2.0 (50). The self-group was defined manually for each analysis, and each threshold cutoff was determined as suggested by the kernel density function for the definition of close and distal group limits. Second, we used a parameter to complement previous analysis, since alterations in codon usage may also suggest HGT. Codon usage may suggest HGT events, since different genomes have differences in the characteristic compositions of the codon usage and GC content. For the codon bias, two different approaches were implemented: raw, directly comparing codon usage in the PKS modules and the genome codon usage; and normalized, comparing the PKS codon usage against the genome codon usage after a normalization accordingly to the amino acid usage of the PKS module. The codon usage was determined using cusp implemented in EMBOSS (51). A χ^2^ test was conducted by using the chisq.test function implemented in R (version 3.3.0) to evaluate the codon usage in the genome and compared with the codon usage observed in the PKS modules separately.

### Accession number(s).

The sequences of the swinholide and scytophycin biosynthesis gene clusters are available at NCBI (https://www.ncbi.nlm.nih.gov/GenBank/) with accession numbers KY767987 and KY767986.

## Supplementary Material

Supplemental material
