# Supplementary material for: The Swinholide Biosynthesis Gene Cluster from a Terrestrial Cyanobacterium, Nostoc sp. Strain UHCC 0450
Source: Appl Environ Microbiol. 2018 Jan 17;84(3):e02321-17. doi: 10.1128/AEM.02321-17 (PMC5772238; doi:10.1128/AEM.02321-17)
Supplement: Supplemental material [file supp_84_3_e02321-17__index.html]

Supplemental material 

# The Swinholide Biosynthesis Gene Cluster from a Terrestrial Cyanobacterium, Nostoc sp. Strain UHCC 0450

## Supplemental material

- Supplemental file 1 -

  Swinholide A through K and isoswinholide A and B structures and exact monoisotopic masses (Fig. S1A); fragmentation analysis of MSE spectra of commercial swinholide A and the compound from *Nostoc* sp. strain UHCC 0450 (Fig. S1B); NMR analysis of the methanol extract of *Nostoc* sp. UHCC 0450 (Fig. S2); chromatograms and product ion spectra of sodiated swinholide molecules produced by *Nostoc* sp. UHCC 0450 (Fig. S3); maximum-likelihood tree of ketosynthase domains 6 to 17 and 19 of the six studied gene clusters (Fig. S4); 1H and 13C NMR data for swinholide A from *Nostoc* sp. UHCC 0450 in D6-DMSO and CD3OD, swinholide A in CD3OD and CDCl3, swinholide F in CDCl3, isoswinholide A in CDCl3, and isoswinholide B in CD3OD (Table S1); retention times, ion masses, and relative intensities of sodiated molecules of swinholide variants 1 to 6 produced by *Nostoc* sp. UHCC 0450 (Table S2); most similar hits in BLASTp searches of the ORFs in the swinholide cluster and surrounding ORFs in *Nostoc* sp. UHCC 0450 (Table S3A); most similar hits in BLASTp searches of the ORFs in the scytophycin cluster and surrounding ORFs in *Anabaena* sp. UHCC 0451 (Table S3B); distance matrices of the six related macrolide biosynthesis gene cluster core genes (Table S4); HGT events detected in HGTector for the PKS modules (Table S5); information used to construct the phylogenetic acyltransferase tree (Table S6).

  PDF, 844K
