# Supplementary material for: The Swinholide Biosynthesis Gene Cluster from a Terrestrial Cyanobacterium, Nostoc sp. Strain UHCC 0450
Source: Appl Environ Microbiol. 2018 Jan 17;84(3):e02321-17. doi: 10.1128/AEM.02321-17 (PMC5772238; doi:10.1128/AEM.02321-17)
Supplement: Supplemental material [file AEM.02321-17_zam003188276s1.pdf]

Supplemental Material

The swinholide biosynthetic gene cluster from a terrestrial cyanobacterium *Nostoc* sp. UHCC 0450

Anu Humisto[1], Jouni Jokela[1], Liwei Liu[1], Matti Wahlsten[1], Hao Wang[1], Perttu Permi[2,3,4], João Paulo Machado[5], Agostinho Antunes[5,6], David P. Fewer[1], Kaarina Sivonen[1]*

[1]Department of Food and Environmental Sciences, Viikki Biocenter 1, University of Helsinki, Helsinki, Finland; [2]Program in Structural Biology and Biophysics, Institute of Biotechnology, University of Helsinki, Helsinki, Finland; [3]Department of Biological and Environmental Science, Nanoscience Center, University of Jyväskylä, Jyväskylä, Finland; [4]Department of Chemistry, Nanoscience Center, University of Jyväskylä, Jyväskylä, Finland; [5]CIIMAR/CIMAR, Interdisciplinary Centre of Marine and Environmental Research, University of Porto, Porto, Portugal; [6]Department of Biology, Faculty of Sciences, University of Porto, Porto, Portugal.

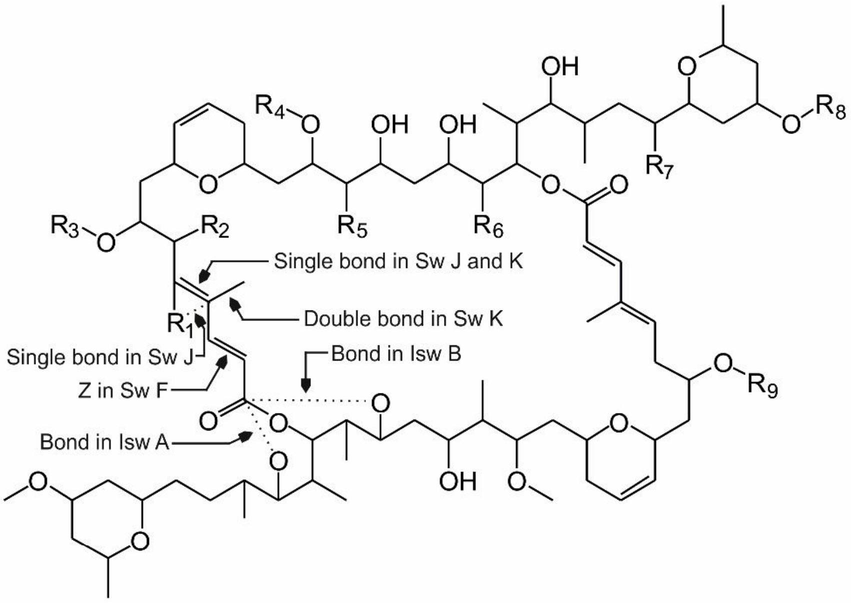

Variant	Exact mass	R_1	R_2	R_3	R_4	R_5	R_6	R_7	R_8	R_9
Sw A	1388.93	H	H	H	CH_3	CH_3	CH_3	H	CH_3	H
Isw A	1388.93	H	H	H	CH_3	CH_3	CH_3	H	CH_3	H
Isw B	1388.93	H	H	H	CH_3	CH_3	CH_3	H	CH_3	H
Sw B	1374.92	H	H	H	CH_3	H	CH_3	H	CH_3	H
Sw C	1374.92	H	H	H	CH_3	CH_3	CH_3	H	H	H
Sw D	1374.92	H	H	H	H	CH_3	CH_3	H	CH_3	H
Sw E	1404.93	H	OH	H	CH_3	CH_3	CH_3	H	CH_3	H
Sw F	1388.93	H	H	H	CH_3	CH_3	CH_3	H	CH_3	H
Sw G	1374.92	H	H	H	CH_3	CH_3	H	H	CH_3	H
Sw H	1416.96	H	H	CH_3	CH_3	CH_3	CH_3	H	CH_3	CH_3
Sw I	1404.93	H	H	H	CH_3	CH_3	CH_3	OH	CH_3	H
Sw J	1404.93	O	H	H	CH_3	CH_3	CH_3	H	CH_3	H
Sw K	1404.93	OH	H	H	CH_3	CH_3	CH_3	H	CH_3	H

FIG S1A Swinholide (Sw) A – K and isoswinholide (Isw) A and B structures and exact monoisotopic masses (Da).

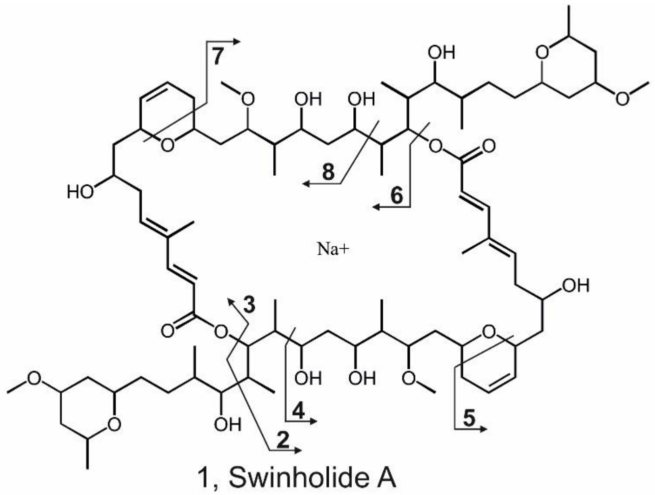

1, Swinholide A

Ion code	Formula	Swinholide A Calc (*m/z*)	Swinholide A Exp (*m/z*)	Swinholide A Δ (ppm)	*Nostoc* sp. 107.3 Exp (*m/z*)	*Nostoc* sp. 107.3 Δ (ppm)
1	$C_{78}H_{132}NaO_{20}^{+}$	1411.9204	1411.9199	-0.4	1411.9187	-1.3
2-7	$C_{65}H_{114}NaO_{17}^{+}$	1189.7948	1189.7923	-2.2	1189.7919	-2.5
3-4	$C_{61}H_{100}NaO_{17}^{+}$	1127.6853	1127.6830	-2.1	1127.6822	-2.8
3-5	$C_{52}H_{84}NaO_{13}^{+}$	939.5804	939.5784	-2.2	939.5777	-2.9
3-6	$C_{39}H_{66}NaO_{10}^{+}$	717.4548	717.4528	-2.9	717.4535	-1.9
6-7	$C_{26}H_{48}NaO_{7}^{+}$	495.3292	495.3292	-0.1	495.3282	-2.2
3-8	$C_{22}H_{34}NaO_{7}^{+}$	433.2197	433.2188	-2.1	433.2183	-3.3

FIG S1B Fragmentation analysis of MS^E spectra (Fig. 1) of commercial swinholide A and the compound from *Nostoc* sp. UHCC 0450.

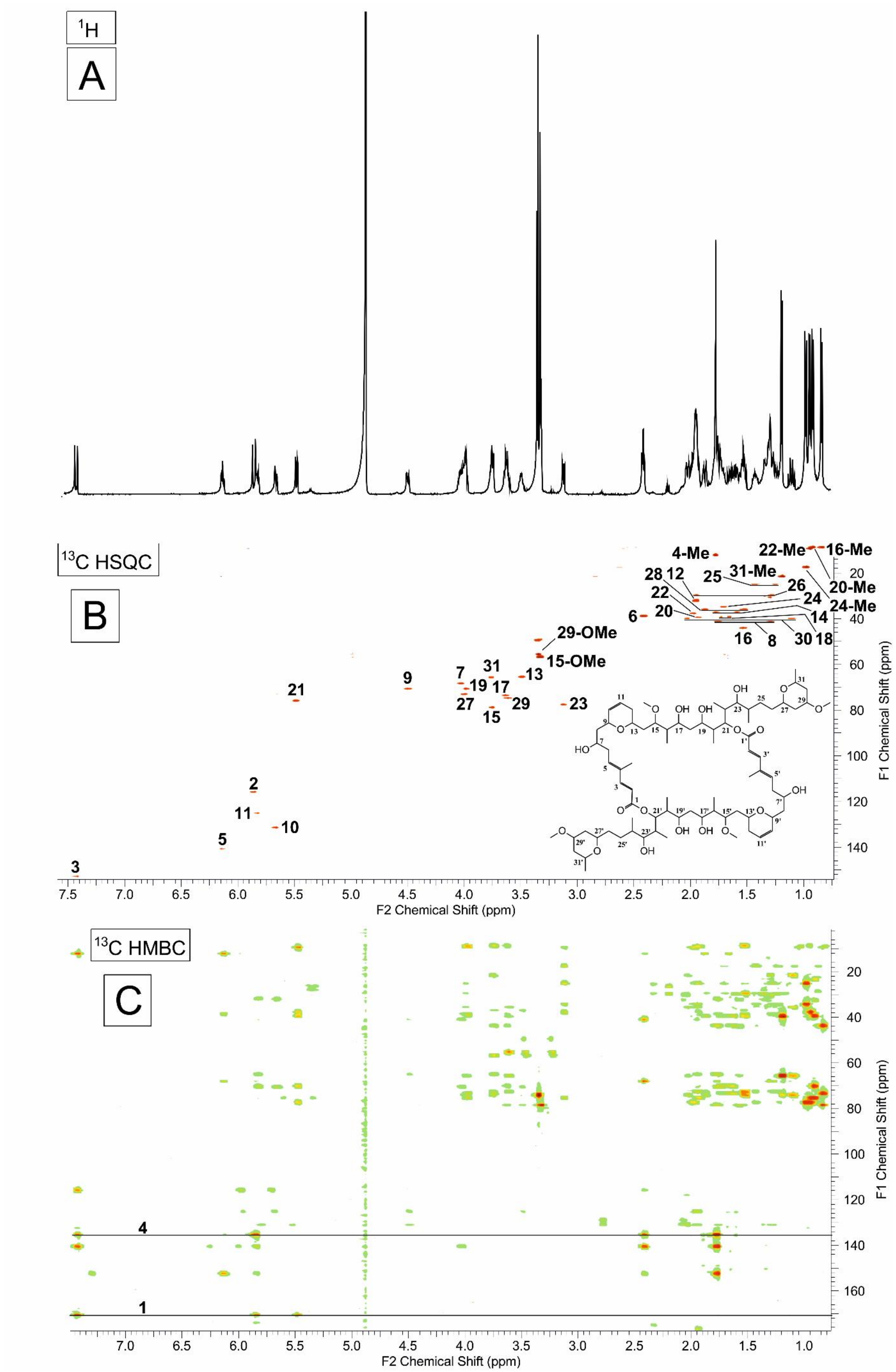

1H
A
13C HSQC
B
13C HMBC
C
F2 Chemical Shift (ppm)
F1 Chemical Shift (ppm)

FIG S2 NMR analysis of methanol extract of *Nostoc* sp. UHCC 0450. A: Proton spectrum, B: ^{13}C HSQC spectrum with annotations (see Table S2) and the carbon numbered structure of swinholide A, C: HMBC spectrum with horizontal lines showing the carbonyl (C1) and quaternary carbon (C4) signals.

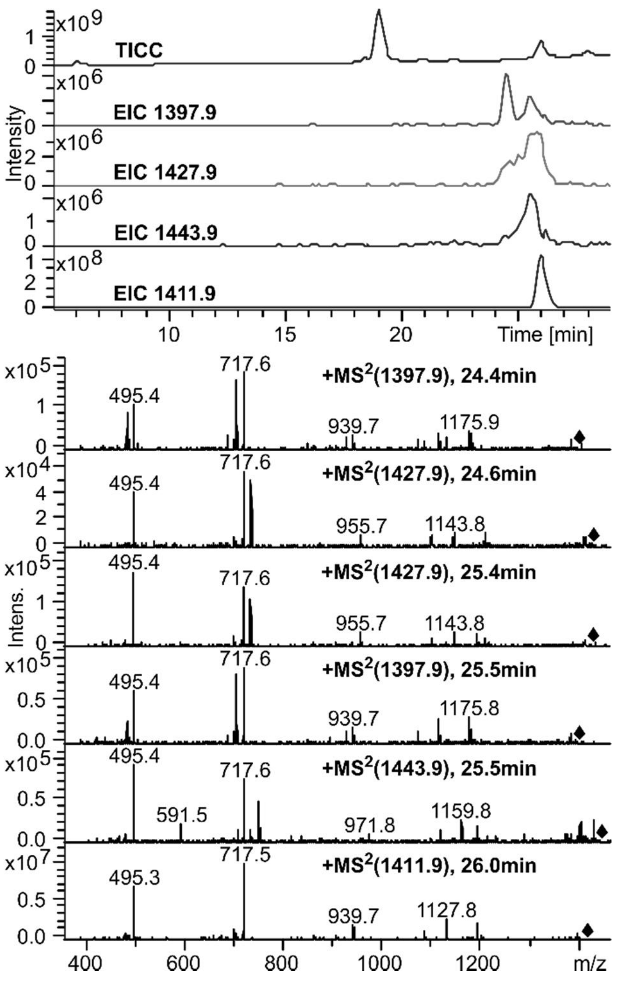

FIG S3 Chromatograms and product ion spectra of sodiated swinholide molecules produced by *Nostoc* sp. UHCC 0450. TICC: total ion current chromatogram, EIC: extracted ion chromatogram, black diamond: sodiated swinholide precursor ion.

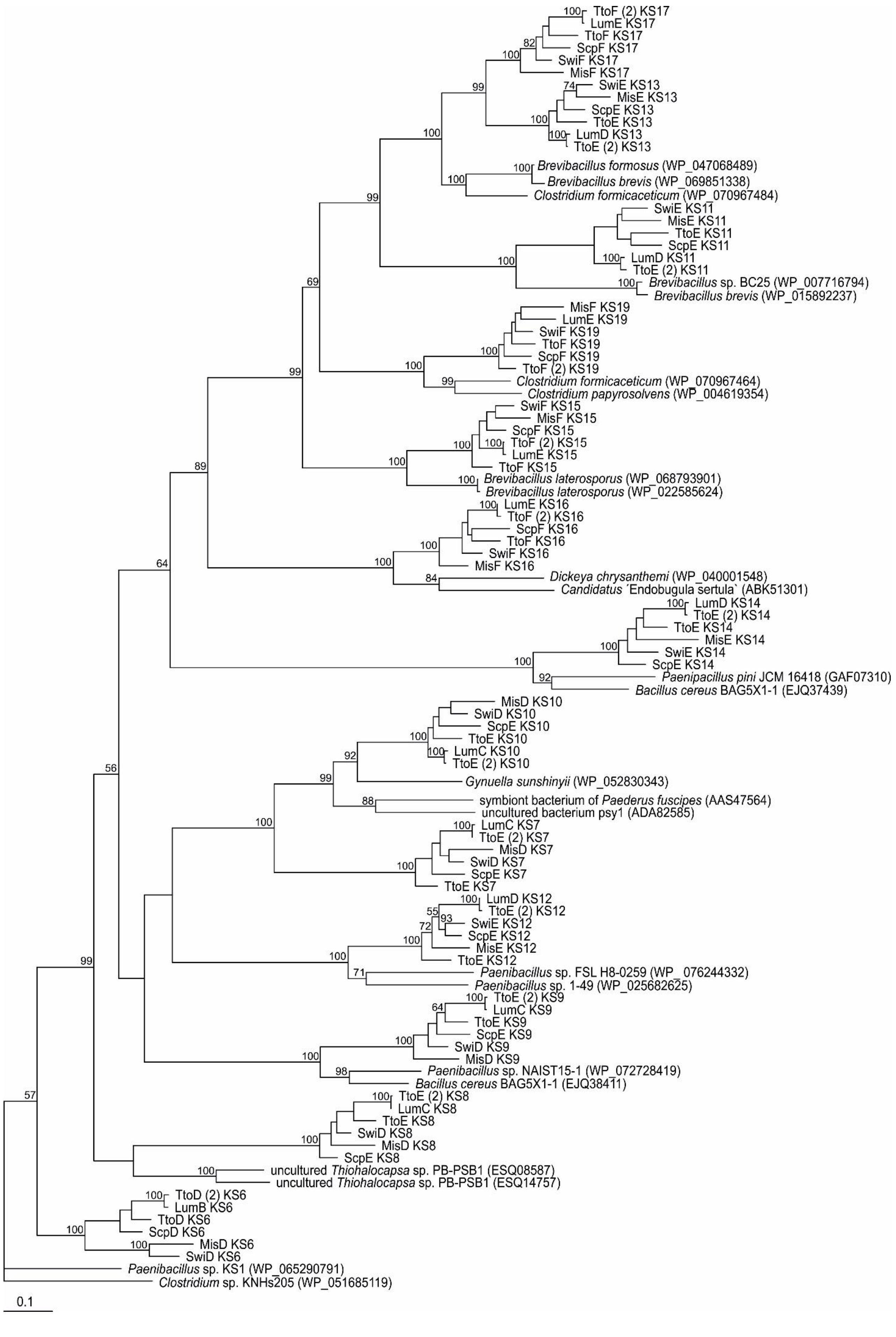

TtoF (2) KS17
LumE KS17
TtoF KS17
ScpF KS17
SwiF KS17
MisF KS17
SwiE KS13
MisE KS13
ScpE KS13
TtoE KS13
LumD KS13
TtoE (2) KS13
Brevibacillus formosus (WP_047068489)
Brevibacillus brevis (WP_069851338)
Clostridium formicaceticum (WP_070967484)
SwiE KS11
MisE KS11
TtoE KS11
ScpE KS11
LumD KS11
TtoE (2) KS11
Brevibacillus sp. BC25 (WP_007716794)
Brevibacillus brevis (WP_015892237)
MisF KS19
LumE KS19
SwiF KS19
TtoF KS19
ScpF KS19
TtoF (2) KS19
Clostridium formicaceticum (WP_070967464)
Clostridium papyrosolvens (WP_004619354)
SwiF KS15
MisF KS15
ScpF KS15
TtoF (2) KS15
LumE KS15
TtoF KS15
Brevibacillus laterosporus (WP_068793901)
Brevibacillus laterosporus (WP_022585624)
LumE KS16
TtoF (2) KS16
ScpF KS16
TtoF KS16
SwiF KS16
MisF KS16
Dickeya chrysanthemi (WP_040001548)
Candidatus ´Endobugula sertula` (ABK51301)
LumD KS14
TtoE (2) KS14
TtoE KS14
MisE KS14
SwiE KS14
ScpE KS14
Paenipacillus pini JCM 16418 (GAF07310)
Bacillus cereus BAG5X1-1 (EJQ37439)
MisD KS10
SwiD KS10
ScpE KS10
TtoE KS10
LumC KS10
TtoE (2) KS10
Gynuella sunshinyii (WP_052830343)
symbiont bacterium of Paederus fuscipes (AAS47564)
uncultured bacterium psy1 (ADA82585)
LumC KS7
TtoE (2) KS7
MisD KS7
SwiD KS7
ScpE KS7
TtoE KS7
LumD KS12
TtoE (2) KS12
SwiE KS12
ScpE KS12
MisE KS12
TtoE KS12
Paenibacillus sp. FSL H8-0259 (WP_076244332)
Paenibacillus sp. 1-49 (WP_025682625)
TtoE (2) KS9
LumC KS9
TtoE KS9
ScpE KS9
SwiD KS9
MisD KS9
Paenibacillus sp. NAIST15-1 (WP_072728419)
Bacillus cereus BAG5X1-1 (EJQ38411)
TtoE (2) KS8
LumC KS8
TtoE KS8
SwiD KS8
MisD KS8
ScpE KS8
uncultured Thiohalocapsa sp. PB-PSB1 (ESQ08587)
uncultured Thiohalocapsa sp. PB-PSB1 (ESQ14757)
TtoD (2) KS6
LumB KS6
TtoD KS6
ScpD KS6
MisD KS6
SwiD KS6
Paenibacillus sp. KS1 (WP_065290791)
Clostridium sp. KNHs205 (WP_051685119)
0.1

FIG S4 Maximum-likelihood tree of ketosynthase (KS) domains 6 to 19, except 18, of the six studied gene clusters. All the similar KS domains from different biosynthetic gene clusters are branched closely together. Bootstrap values from the neighbor-joining tree (1000 replicates) were attached into the maximum-likelihood tree. Only matching bootstrap values over 50 are represented. Two outgroup sequences from each ketosynthases were added.

Table S1 ^{1}H and ^{13}C NMR data (ppm) for swinholide A from *Nostoc* sp. UHCC 0450 in d6-DMSO and CD_3OD, swinholide A (A) in CD_3OD and $CDCl_3$, Swinholide F (F) in $CDCl_3$, isoswinholide A (Iso A) in $CDCl_3$ and isoswinholide B (Iso B) in CD_3OD. Yellow and green columns highlight the δ_C and δ_H values of swinholide A from *Nostoc* sp. UHCC 0450 and *Theonella swinhoei* from Solomon Islands in CD_3OD. Grey rows highlight the δ_C and δ_H values which are the most specific for the different swinholides which have the same elemental composition (swinholides A and F and isoswinholides A and B).

**Reference:**	***Nostoc* sp. UHCC 0450 Swinholide A**				De Marino 2011		Andrianasolo 2005		Youssef 2006		Tsukamoto 1991		Kobayashi 1990		Sinisi 2013			
	**d6-DMSO**		**CD_3OD**		**A in CD_3OD**		**A in $CDCl_3$**		**A in $CDCl_3$**		**F in $CDCl_3$**		**Iso A in $CDCl_3$**		**Iso B in CD_3OD**			
**position**	δ_C	δ_H	δ_C	δ_H	δ_C	δ_H	δ_C	δ_H	δ_C	δ_H	δ_H	$\delta_{H'}$	δ_H	$\delta_{H'}$	δ_C	δ_H	$\delta_{C'}$	$\delta_{H'}$
1	167.4		170.3		170.6		170.1		170.0						169.4		170.3	
2	114.5	5.76	115.9	5.85	115.6	5.84	113.3	5.79	113.2	5.79	5.80	5.71	5.85	5.84	115.0	5.86	115.0	5.89
3	150.0	7.26	152.6	7.42	152.3	7.43	153.3	7.58	153.2	7.58	7.56	6.55	7.45	7.40	149.9	7.36	149.9	7.44
4	132.9		135.4		135.5		134.3		134.2						135.1		135.1	
4-Me	11.4	1.69	12.4	1.77	12.4	1.77	12.3	1.81	12.3	1.88	1.81	1.84	1.84	1.82	11.4	1.80	11.4	1.80
5	140.2	6.12	140.5	6.13	140.5	6.14	142.3	6.08	142.2	6.08	6.13	5.88	6.10	6.11	139.3	6.06	139.1	6.18
6	37.1	2.20	38.9	2.42	38.8	2.40	37.4	2.17	37.4	2.46	2.25		2.37		37.5	2.38	37.5	2.43
6'		2.37						2.46		2.18	2.46	2.32	2.45	2.37				
7	66.0	3.81	68.1	4.02	68.1	4.02	66.7	4.16	66.6	4.14	4.15		4.01	4.11	67.2	4.00	66.7	4.11
8	39.5	1.21	41.1	1.29	41.0	1.28	41.1	1.60	40.8	1.58	1.56	1.47	1.45	1.50	40.0	1.37	40.0	1.33
8'		1.61		1.76		1.76		-		1.73	1.63	1.76	1.65			1.75		1.76
9	68.4	4.34	70.7	4.49	70.5	4.47	65.9	4.52	65.7	4.51	4.52		4.50	4.52	69.1	4.47	69.1	4.47
10	130.8	5.64	131.1	5.66	130.9	5.65	129.9	5.69	129.8	5.69	5.68	5.65	5.68		129.8	5.66	129.8	5.66
11	123.8	5.75	125.2	5.82	124.9	5.81	123.3	5.78	123.2	5.78	5.77	5.81	5.80		123.6	5.82	123.6	5.82
12	30.8	1.87	32.1	1.95	32.2	1.94	30.0	1.89	29.9	1.82	1.84		1.92	1.93	31.6	1.96	31.1	2.01
12'								2.28		2.27	2.21		2.03	2.08				
13	63.0	3.37	65.4	3.49	65.3	3.49	65.8	3.90	65.8	3.86	3.84		3.73	3.70	63.9	3.55	64.4	3.60

14	35.6	1.43	37.3	1.58	37.2	1.58	33.9	1.45	33.8	1.46	1.54		1.47	1.52	37.2	1.57	35.7	1.59
14'		1.66		1.77		1.77		2.15		2.14	2.10		1.98	1.96		1.83		1.87
15	76.2	3.61	78.5	3.75	78.2	3.76	75.1	4.01	75.1	4.01	3.92		3.71	3.73	78.1	3.77	77.3	3.79
15-OMe	55.9	3.18	57.0	3.32	56.7	3.32	57.5	3.35	57.4	3.35	3.35	3.38	3.36	3.38	55.8	3.33	55.8	3.35
16	41.9	1.41	43.9	1.53	43.8	1.52	41.1	1.68	41.0	1.68	1.68	1.60	1.63		43.9	1.53	41.9	1.59
16-Me	8.3	0.71	8.7	0.84	8.8	0.83	9.1	0.81	9.4	0.81	0.81	0.79	0.79	0.81	8.4	0.84	8.4	0.85
17	70.0	3.36	73.5	3.62	73.2	3.61	73.9	3.84	73.8	3.83	3.76		3.77	3.75	73.1	3.62	69.8	3.61
18	38.0	1.43	39.1	1.66	39.0	1.63	38.5	1.58	38.4	1.62	1.48		1.50		39.1	1.60	38.3	1.83
18'		1.59		1.73		1.74		1.63			1.50		1.50	1.55		1.76		1.88
19	66.9	3.76	70.4	3.97	70.1	3.97	71.4	4.01	71.3	3.98	3.92		4.25	3.80	71.9	3.99	74.2	5.65
20	37.6	1.80	39.5	1.94	39.4	1.94	40.9	1.75	41.3	1.75	1.71	1.72	1.66	1.75	39.3	1.91	36.9	1.98
20-Me	8.3	0.76	9.0	0.91	8.9	0.91	9.4	0.98	9.2	0.97	0.91	0.93	0.81	0.90	13.2	0.91	8.6	0.94
21	73.9	5.34	75.7	5.48	75.6	5.46	74.4	5.35	74.3	5.36	5.36	5.30	3.56	5.34	75.1	5.40	73.0	3.64
22	36.7	1.83	38.0	1.98	37.9	1.98	37.7	1.93	37.6	1.95	1.89		1.90	1.88	37.7	1.93	37.9	1.85
22-Me	9.1	0.80	9.6	0.94	9.6	0.94	9.2	0.83	9.1	0.84	0.83	0.84	0.89	0.87	8.8	0.94	8.8	0.95
23	74.7	2.91	77.5	3.12	77.2	3.11	76.0	3.13	76.0	3.12	3.11	3.04	4.91	3.07	76.2	3.12	78.2	3.36
24	32.5	1.56	34.5	1.71	34.4	1.70	33.3	1.66	33.2	1.65	1.63	1.68	1.93	1.66	34.6	1.73	33.0	1.67
24-Me	16.9	0.86	17.7	0.98	17.7	0.98	17.8	0.99	17.7	0.99	0.97	0.99	0.93	0.99	16.7	0.98	16.1	0.93
25	23.9	1.08	25.2	1.25	25.1	1.24	24.0	1.26	23.9	1.27	1.27		1.33	1.26	24.2	1.28	24.2	1.28
25'		1.26		1.43		1.42		1.38		1.38	1.39		1.48	1.37		1.42		1.42
26	27.8	1.14	29.9	1.29	29.7	1.27	29.4	1.25	29.3	1.30	1.26		1.22		29.0	1.30	29.0	1.30
26'		1.80		1.94		1.94		1.87		1.90	1.88		1.86	1.87		1.94		1.94
27	70.0	3.85	72.9	4.00	72.7	3.99	71.4	4.01	71.4	4.02	4.00		4.01		72.4	3.97	72.4	3.97
28	34.1	1.38	35.8	1.52	35.8	1.52	34.9	1.59	34.8	1.60	1.58		1.57		35.7	1.53	35.7	1.53
28'		1.74		1.86		1.87		1.82		1.82	1.81		1.78			1.87		1.87
29	72.3	3.49	74.4	3.60	74.2	3.61	73.3	3.54	73.2	3.53	3.53		3.52		74.1	3.63	74.1	3.63
29-OMe	54.3	3.21	55.4	3.34	55.3	3.34	55.3	3.33	55.2	3.33	3.34		3.35	3.34	54.2	3.34	54.2	3.34
30	38.0	0.96	39.8	1.09	39.7	1.09	38.7	1.17	38.8	1.18	1.18		1.16		39.5	1.11	39.5	1.11
30'		1.92		2.01		2.01		1.97		1.96	1.98		1.94			1.91		1.91
31	63.7	3.60	65.8	3.75	65.7	3.74	64.6	3.70	64.5	3.69	3.69		3.67	3.69	65.7	3.76	65.7	3.76
31-Me	21.5	1.08	21.8	1.19	21.8	1.19	21.8	1.20	21.7	1.20	1.20		1.20		20.8	1.19	20.8	1.19

Table S2 Swinholide variants 1 – 6, retention times (min), ion masses and relative intensities (RI, %) of sodiated molecules produced by *Nostoc* sp. UHCC 0450. Data measured with LC-ITMS.

Variant	t_R (min)	$[M+Na]^+$ (m/z)	Monoisotopic mass	RI (%)
1	24.4	1397.9	1374.9	3.1
2	24.6 - 25.0	1427.9	1404.9	2.7
3	25.4	1443.9	1420.9	2.7
4	25.5	1397.9	1374.9	2.0
5	25.5 - 25.7	1427.9	1404.9	5.5
6, Swh A	25.9	1411.9	1388.9	84

Table S3A Most similar hits in BLASTp searches of the ORFs in swinholide cluster and surrounding ORFs in *Nostoc* sp. UHCC 0450. Lengths of amino acid sequences (size), sequence directions (reverse/forward), identity (id) and query coverage (QC) values and NCBI accession numbers are listed.

ORF	*Size (aa)*	*Seq*	*Most similar hit in BLASTp search*	*Id/QC*	*Accession number*
Orf1 - SwiC	6099	*rev*	Malonyl CoA-acyl carrier protein transacylase (*Candidatus* ´Entotheonella` sp. (ex. *Theonella swinhoei*) (MisC)	73/100	AKQ22699.1
Orf2 - SwiD	9161	*for*	Malonyl CoA-acyl carrier protein transacylase (*Candidatus* ´Entotheonella` sp. (ex. *Theonella swinhoei*) (MisD)	73/98	AKQ22698.1
Orf3 - SwiE	4462	*for*	Malonyl CoA-acyl carrier protein transacylase (*Scytonema* sp. PCC 10023) (TtoE)	78/99	AKQ22650.1
Orf4 - SwiF	8017	*for*	Malonyl CoA-acyl carrier protein transacylase (*Scytonema* sp. PCC 10023) (TtoF)	79/99	AKQ22649.1
Orf5 - SwiG	374	*for*	Malonyl-CoA-acyl carrier protein transacylase (*Scytonema* sp. PCC 10023) (TtoA)	89/95	AKQ22643.1
Orf6	228	*for*	Hypothetical protein (*Tolypothrix* sp. PCC 7601)	87/93	WP_045874996.1
Orf7	327	*for*	Hypothetical protein (*Nodularia spumigena*)	86/98	WP_063873654.1
Orf8	1538	*for*	WD-40 repeat protein (*Lyngbya* sp. PCC 8106)	64/99	WP_009785093.1
Orf9	106	*rev*	Hypothetical protein (*Nostoc punctiforme*)	81/84	WP_012411093.1
Orf10	3832	*rev*	NpnC (*Nostoc* sp. 152)	72/99	AEU11003.1
Orf11	1641	*rev*	Type I PKS (*Fischerella* sp. PCC 9339)	63/100	WP_017308564.1
Orf12	119	*rev*	Hypothetical protein (*Nostoc* sp. 'Peltigera membranacea cyanobiont')	74/94	AGH69799.1
Orf13	473	*for*	Phosphomethylpyrimidine synthase (*Nostoc punctiforme*)	98/96	WP_012410061.1
Orf14	271	*for*	Hypothetical protein (*Scytonema hofmanni,* UTEX B 1581)	92/95	WP_029637529.1
Orf15	663	*rev*	Peptidase S8 (*Nostoc punctiforme*)	90/99	WP_012410060.1
Orf16	350	*for*	Hypothetical protein (*Nostoc punctiforme*)	91/98	WP_012408528.1
Orf17	139	*rev*	Hypothetical protein (*Nostoc punctiforme*)	90/99	WP_012412679.1
Orf18	329	*rev*	Deoxyribodipyrimidine photo-lyase (*Nostoc punctiforme*)	90/86	WP_012412678.1
Orf19	457	*for*	Hypothetical protein (Oscillatoriales cyanobacterium MTP1)	44/66	WP_058882336.1
Orf20	375	*for*	Histidinol-phosphate transaminase (*Nostoc punctiforme*)	90/94	WP_012407529.1
Orf21	288	*rev*	Universal stress protein UspA (*Nostoc punctiforme*)	95/98	WP_012407090.1
Orf22	563	*rev*	Alkaline phosphatase (*Nostoc punctiforme*)	90/97	WP_012407091.1

Table S3B Most similar hits in BLASTp searches of the ORFs in scytophycin cluster and surrounding ORFs in *Anabaena* sp. UHCC 0451. Lengths of amino acid sequences (size), sequence directions (reverse/forward), identity (id) and query coverage (QC) values and NCBI accession numbers are listed.

ORF	***Size (aa)***	***Seq***	***Most similar hit in BLASTp search***	***Id/QC***	***Accession number***
Orf1	324	*rev*	1,4-dihydroxy-2-naphthoyl-CoA synthase (*Trichormus* sp. NMC-1)	96/85	WP_071188441.1
Orf2	103	*rev*	Hypothetical protein AN483 20245 (*Aphanizomenon flos-aquae* MDT14a)	93/93	OBQ27510.1
Orf3	420	*rev*	Glycosyl transferase family 1 (*Trichodesmium erythraeum*)	68/91	WP_011613192.1
Orf4 - ScpB	290	*rev*	C5-O-methyltransferase (*Scytonema* sp. PCC 10023)	85/100	AKQ22642.0
Orf5 - ScpA	354	*rev*	Malonyl CoA-ACP transacylase (*Aphanizomenon flos-aquae* MDT14a)	90/98	OBQ27524.1
Orf6	595	*rev*	Protein kinase (*Aphanizomenon flos-aquae* MDT14a)	93/100	OBQ27523.1
Orf7	319	*for*	Hypothetical protein (*Moorea producens*)	57/94	WP_070395343.1
Orf8	2269	*for*	Polyketide synthase module (*Planktothrix tepida*)	72/97	WP_072719775.1
Orf9	1933	*for*	Putative 3-oxoacyl-(acyl-carrier-protein) reductase (*Planktothrix sert*a PCC 8927)	76/96	CUR19796.1
Orf10	1070	*for*	Type I polyketide synthase (*Tolypothrix* sp. PCC 7601)	79/99	WP_052335497.1
Orf11	1563	*for*	Hypothetical protein (*Anabaena* sp. PCC 7108)	51/52	WP_016949096.1
Orf12 - ScpC	4940	*rev*	Malonyl CoA-acyl carrier protein transacylase (*Scytonema* sp. PCC 10023) (TtoC)	79/99	AKQ22652.1
Orf13 - ScpD	5040	*rev*	Malonyl CoA-acyl carrier protein transacylase (*Scytonema* sp. PCC 10023) (TtoD)	77/99	AKQ22651.1
Orf14 - ScpE	10337	*rev*	Malonyl CoA-acyl carrier protein transacylase (*Scytonema* sp. PCC 10023) (TtoE)	76/99	AKQ22650.1
Orf15 - ScpF	7957	*rev*	Malonyl CoA-acyl carrier protein transacylase (*Scytonema* sp. PCC 10023) (TtoF)	78/100	AKQ22649.1
Orf16 - ScpG	458	*rev*	Cytochrome P450 (*Scytonema* sp. PCC 10023) (TtoG)	92/100	AKQ22648.1
Orf17	310	*rev*	Protein involved in biosynthesis of mitomycin antibiotics/polyketide fumonisin (*Planktothrix* sp. PCC 11201)	92/99	CUR23620.1
Orf18	298	*rev*	Protein involved in biosynthesis of mitomycin antibiotics/polyketide fumonisin (*Planktothrix* sp. PCC 11201)	92/98	CUR23622.1

Table S4 Distance matrices of the six related macrolide biosynthetic gene cluster core genes. Nucleotide and amino acid sequence identities are presented as percentages.

	Base Identity (%)							Amino acid Identity (%)					
	swiC	*misC*	*scpC*	*ttoC*	*ttoC (2)*	*lumA*		SwiC	MisC	ScpC	TtoC	TtoC (2)	LumA
swiC	100.0						SwiC	100.0					
misC	79.1	100.0					MisC	75.7	100.0				
scpC	42.9	42.7	100.0				ScpC	23.3	22.9	100.0			
ttoC	43.2	43.2	82.3	100.0			TtoC	23.0	22.8	79.6	100.0		
ttoC (2)	43.6	43.1	79.1	80.9	100.0		Tto2C	23.3	22.9	77.1	79.4	100.0	
lumA	43.8	43.2	79.1	80.7	97.5	100.0	LumA	23.5	23.0	77.0	79.0	96.1	100.0
	swiD	*misD*	*scpD*	*ttoD*	*ttoD (2)*	*lumB*		SwiD	MisD	ScpD	TtoD	TtoD (2)	LumB
swiD	100.0						SwiD	100.0					
misD	78.7	100.0					MisD	75.2	100.0				
scpD	44.6	45.1	100.0				ScpD	26.6	26.4	100.0			
ttoD	44.4	45.0	80.3	100.0			TtoD	25.7	25.9	79.3	100.0		
ttoD (2)	45.4	45.8	79.1	79.6	100.0		Tto2D	27.0	26.6	77.0	78.6	100.0	
lumB	45.1	45.5	79.3	79.8	97.7	100.0	LumB	26.4	26.2	77.4	79.2	97.1	100.0
	swiE	*misE*	*scpE*	*ttoE*	*ttoE (2)*	*lumD*		SwiE	MisE	ScpE	TtoE	TtoE (2)	LumD
swiE	100.0						SwiE	100.0					
misE	79.0	100.0					MisE	78.0	100.0				
scpE	84.4	76.1	100.0				ScpE	82.4	74.7	100.0			
ttoE	82.1	75.5	79.9	100.0			TtoE	80.6	74.5	78.5	100.0		
ttoE (2)	79.8	73.4	78.4	77.7	100.0		Tto2E2	78.8	72.6	76.6	75.8	100.0	
lumD	79.8	73.4	78.3	77.6	98.9	100.0	LumD	78.7	72.3	76.4	75.6	98.4	100.0
	swiF	*misF*	*scpF*	*ttoF*	*ttoF (2)*	*lumE*		SwiF	MisF	ScpF	TtoF	TtoF (2)	LumE
swiF	100.0						SwiF	100.0					
misF	79.5	100.0					MisF	75.3	100.0				
scpF	84.1	75.5	100.0				ScpF	81.4	71.9	100.0			
ttoF	83.9	76.1	81.0	100.0			TtoF	81.1	72.5	79.2	100.0		
ttoF (2)	80.6	73.1	79.8	79.4	100.0		Tto2F	78.4	70.4	77.7	78.0	100.0	
lumE	69.4	64.0	68.3	68.1	81.7	100.0	LumE	79.5	71.2	76.4	77.5	91.1	100.0
	swiG	*misG*	*scpA*	*ttoA*	*ttoA (2)*	*lumJ*		SwiG	MisG	ScpA	TtoA	TtoA (2)	LumJ
swiG	100.0						SwiG	100.0					
misG	83.0	100.0					MisG	84.4	100.0				
scpA	88.3	80.3	100.0				ScpA	83.8	78.8	100.0			
ttoA	90.6	82.1	88.7	100.0			TtoA	87.6	81.9	83.8	100.0		
ttoA (2)	85.1	78.1	84.2	86.8	100.0		Tto2A	81.5	76.7	78.7	84.9	100.0	
lumJ	85.5	78.5	84.5	87.2	98.4	100.0	LumJ	82.7	77.6	79.8	86.1	98.5	100.0

Table S5 HGT events detected in HGTector for the PKS modules. Length:Query length; Hits: BLAST hits of each query group; Self: Query genome or its immediate sister organisms; Close: Other species of the same genus or other genera of the same family, which the query genome belongs; Distal: Phylogenetically distant organisms from the query genome; HGT: Horizontal gene transfer; POE: Putative ORFans or annotation errors.

Query		**Length**	**Hits**	**Self**	**Close**	**Distal**	**HGT**	**POE**	**Best distal match**	**Putative donor group**
swinholide	swiC	6099	1	1	0	0		1		
	swiD	9161	1	1	0	0		1		
	swiE	4462	5	1	0.77	0.36			*Candidatus Profftella armatura*	
	swiF	8017	1	1	0	0		1		
	swiG	374	200	1	0.8	69.41			*Chromobacterium sp. 14B-1*	
misakinolide	misC	5957	1	1	0	0		1		
	misD	8993	4	1	0	0.41			*Burkholderia sp. b14*	
	misE	4508	60	1	0	8.61	1		*Planktothrix paucivesiculata PCC 9631*	Oscillatoriales
	misF	8016	11	1	0	1.34			*Paraburkholderia rhizoxinica HKI 454*	
	misG	354	403	1	0	125.33	1		*Planktothrix paucivesiculata PCC 9631*	Oscillatoriales
scytophycin	ScpC	4941	8	1	0	0.73	1		*Actinomyces israelii DSM 43320*	Actinomycetales
	scpA	355	435	1	0.82	133.65			*Methylococcaceae bacterium Sn10-6*	
	scpD	5041	21	1	0	2.28	1		*Thioploca ingrica*	Thiotrichales
	scpE	10338	2	1	0.33	0				
	scpF	7958	12	1	0	1.53	1		*Paraburkholderia rhizoxinica HKI 454*	Burkholderiales
luminaolide	lumB	5032	6	1	0	0.66			*Paraburkholderia rhizoxinica HKI 454*	
	LumC	5775	1	1	0	0		1		
	lumA	4880	1	1	0	0		1		
	lumD	4503	8	1.99	0	0.95			*Bacillus sp. GZB*	
	lumE	5707	1	1	0	0		1		
	lumJ	406	200	1.87	0	62.68	1		*Kordia zhangzhouensis*	Flavobacteriales
tolytoxin 1	ttoA	356	200	1	0.87	71.76			*Chromobacterium sp. 14B-1*	
	ttoC	5014	1	1	0	0		1		
	ttoD	5113	7	1	0	0.82	1		*Thioploca ingrica*	Thiotrichales
	ttoE	10481	1	1	0	0		1		
	ttoF	8106	1	1	0	0		1		
tolytoxin 2	tto2A	353	200	1	0.99	72.04			*Kordia zhangzhouensis*	
	tto2C	4882	1	1	0	0		1		
	tto2D	4747	5	1	0	0.59	1		*Paraburkholderia rhizoxinica HKI 454*	Burkholderiales
	tto2E	5771	3	1	0	0.31			*Bacillus atrophaeus subsp. globigii*	
	tto2E2	4503	9	1	0.98	1.13			*Bacillus sp. GZB*	
	tto2F	7946	7	1	0	0.73	1		*Sphingomonas sp. CCH16-B10*	Sphingomonadales

Table S6 Information used to construct phylogenetic acyltransferase tree: compounds produced by the PKS cluster, producing strain, *trans*-AT protein and the NCBI accession number.

Compound	Strain information	Protein	Accession number	Reference
9-methylstreptimidone	*Streptomyces himastatinicus* ATCC 53653	SmdF	CCC21120.1	Wang et al. 2013
Bacillaene	*Bacillus velezensis* FZB42	BaeC	CAG23950.2	Chen et al. 2006
Basiliskamide	*Brevibacillus laterosporus* PE36	BasH	ERM18800.1	Theodore et al. 2014
Bongkretic acid	*Burkholderia gladioli* DMSZ11318	BonK	AFN27477.1	Moebius et al. 2012
Bryostatin	*Candidatus* ´Endobugula sertula` (*Bugula neritina* symbiont)	BryP	ABK51299.2	Hildebrand et al. 2004
Calyculin	Uncultured *Candidatus* ´Entotheonella` sp.	CalY	BAP05573.1	Wakimoto et al. 2014
Chivosazole	*Sorangium cellulosum* So ce56	ChiA	AAY89048.1	Perlova et al. 2006
Cycloheximide / actiphenol	*Streptomyces* sp. YIM 56141	ChxB	AFO59863.1	Yin et al. 2014
Diaphorin	*Candidatus* ´Profftella armatura` sp.	DipA	AGS06838.1	Nakabachi et al. 2013
Difficidin	*Bacillus velezensis* FZB42	DifA	CAG23974.1	Chen et al. 2006
Disorazole	*Sorangium cellulosum* So ce12	DszD	AAY32968.1	Carvalho et al. 2005
Dorrigocin / migrastatin	*Streptomyces platensis* sp. rosaceus NRRL 18993	MgsH	ACY01393.1	Lim et al. 2009
Elansolid	*Chitinophaga sancti*	ElaB	AEC04348.1	Dehn et al. 2011
FR901464	*Pseudomonas* sp. 2663	Fr9J	ADH01491.1	Zhang et al. 2011
FR901464	*Pseudomonas* sp. 2663	Fr9O	ADH01496.1	Zhang et al. 2011
Griseoviridin / viridogrisein	*Streptomyces griseoviridis* NRRL 2427	SgvQ	AGN74897.1	Xie et al. 2012
Kalimantacin/batumin	*Pseudomonas fluorescens* BCCM_ID9359	BatJ	ADD82951.1	Mattheus et al. 2010
Kirromycin	*Streptomyces collinus* Tu 365	KirCI	CAN89639.1	Weber et al. 2008
Lactimidomycin	*Streptomyces amphibiosporus* ATCC 53964	LtmH	ACY01403.1	Seo et al. 2014
Leinamycin	*Streptomyces atroolivaceus* S-140	LnmG	AAN85520.1	Cheng et al. 2002
Luminaolide	*Planktothrix paucivesiculata* PCC 9631	LumJ	AKQ22662.1	Ueoka et al. 2015
Macrolactin	*Bacillus velezensis* FZB42	MlnA	CAG23963.1	Schneider et al. 2007
Malleilactone / burkholderic acid	*Burkholderia thailandensis* E264	MalL	ABC35574.1	Biggins et al. 2012
Misakinolide	*Candidatus* ´Entotheonella` sp. TSWA-1 (ex. *Theonella swinhoei*)	MisG	AKQ22695.1	Ueoka et al. 2015
Mupirocin	*Pseudomonas fluorescens* NCIMB 10586	MmpC	AAM12912.1	El-Sayed et al. 2003
Myxopyronin	*Myxococcus fulvu*s Mx f50	MxnA	AGS77281.1	Sucipto et al. 2013

Myxovirecin	*Myxococcus xanthus* DK 1622	TaV	ABF91606.1	Simunovic et al. 2006
Nosperin	*Nostoc* sp. 'Peltigera membranacea cyanobiont'	NspK	ADA69247.1	Kampa et al. 2013
Oocydin	*Serratia marcescens* MSU97	OocV	AFX60321.1	Matilla et al. 2012
Oocydin	*Serratia marcescens* MSU97	OocW	AFX60322.1	Matilla et al. 2012
Oxazolomycin	*Streptomyces albus* JA3453	OzmM	ABS90474.1	Zhao et al. 2006
Patellazole	*Candidatus* ´Endolissoclinum faulkneri` sp. L2	PtzL	AFX98844.1	Kwan et al. 2012
Pederin	Symbiont bacterium of *Paederus fuscipes*	PedD	AAS47563.1	Piel et al. 2002
Phormidolide	*Leptolyngbya* sp. ISBN3-Nov-94-8	PhmD	AMH40436.1	Bertin et al. 2016
Pristinamycin	*Streptomyces pristinaespiralis* Pr11	SnaM	CBW45739.1	Mast et al. 2011
Psymberin	Uncultured bacterium psy1 (sponge *Psammocinia* aff. *Bulbosa)*	PsyH	ADA82589.1	Fisch et al. 2009
Rhizopodin	*Stigmatella aurantiaca* Sg a15	RizA	CCA89325.1	Pistorius et al. 2012
SIA7248	*Streptomyces* sp. A7248	SiaB1	AFS33444.1	Zou et al. 2013
Sorangicin	*Sorangium cellulosum* So ce12	SorO	ADN68489.1	Irschik et al. 2010
Tartrolon	*Teredinibacter turnerae* T7901	TrtB	ACR11036.1	Elshahawi et al. 2013
Thailandamide	*Burkholderia thailandensis* E264	TaiC	ABC34740.1	Nguyen et al. 2008
Thailanstatin	*Burkholderia thailandensis* MSMB43	TstJ	AGN11884.1	Liu et al. 2013
Thailanstatin	*Burkholderia thailandensis* MSMB43	TstO	AGN11889.1	Liu et al. 2013
Thiomarinol	*Pseudoalteromonas* sp. SANK 73390	TmpC	CBK62732.1	Fukuda et al. 2011
Tolytoxin	*Scytonema* sp. PCC 10023	TtoA	AKQ22643.1	Ueoka et al. 2015
Tolytoxin	*Planktothrix* sp. PCC 11201	TtoA (2)	CUR23626.1	Pancrace et al. 2017
Virginiamycin	*Streptomyces virginiae* MAFF10-06014	VirI	BAF50719.1	Pulsawat et al. 2007
Enacyloxin	*Burkholderia ambifaria* AMMD	(AT1, cis)	ABI91466.1	Mahenthiralingam et al. 2011

References for Table S6

1. Wang B, Song Y, Luo M, Chen Q, Ma J, Huang H, Ju J. 2013. Biosynthesis of 9-methylstreptimidone involves a new decarboxylative step for polyketide terminal diene formation. Org Lett 15:1278–1281.

2. Chen X-H, Vater J, Piel J, Franke P, Scholz R, Schneider K, Koumoutsi A, Hitzeroth G, Grammel N, Strittmatter AW, Gottschalk G, Süssmuth RD, Borriss R. 2006. Structural and functional characterization of three polyketide synthase gene clusters in *Bacillus amyloliquefaciens* FZB 42. J Bacteriol 188:4024–36.

3. Theodore CM, Stamps BW, King JB, Price LSL, Powell DR, Stevenson BS, Cichewicz RH. 2014. Genomic and metabolomic insights into the natural product biosynthetic diversity of a feral-hog-associated *Brevibacillus laterosporus* strain. PLoS One 9:3–12.

4. Moebius N, Ross C, Scherlach K, Rohm B, Roth M, Hertweck C. 2012. Biosynthesis of the respiratory toxin bongkrekic acid in the pathogenic bacterium *Burkholderia gladioli*. Chem Biol 19:1164–1174.

5. Hildebrand M, Waggoner LE, Liu H, Sudek S, Allen S, Anderson C, Sherman DH, Haygood M. 2004. bryA: An unusual modular polyketide synthse gene from the uncultivated bacterial symbiont of the marine bryozoan *Bugula neritina*. Chem Biol 11:1543–1552.

6. Wakimoto T, Egami Y, Nakashima Y, Wakimoto Y, Mori T, Awakawa T, Ito T, Kenmoku H, Asakawa Y, Piel J, Abe I. 2014. Calyculin biogenesis from a pyrophosphate protoxin produced by a sponge symbiont. Nat Chem Biol 10:648–655.

7. Perlova O, Gerth K, Kaiser O, Hans A, Müller R. 2006. Identification and analysis of the chivosazol biosynthetic gene cluster from the myxobacterial model strain *Sorangium cellulosum* So ce56. J Biotechnol 121:174–191.

8. Yin M, Yan Y, Lohman JR, Huang SX, Ma M, Zhao GR, Xu LH, Xiang W, Shen B. 2014. Cycloheximide and actiphenol production in *Streptomyces* sp. YIM56141 governed by single biosynthetic machinery featuring an acyltransferase-less type I polyketide synthase. Org Lett 16:3072–3075.

9. Nakabachi A, Ueoka R, Oshima K, Teta R, Mangoni A, Gurgui M, Oldham NJ, Van Echten-Deckert G, Okamura K, Yamamoto K, Inoue H, Ohkuma M, Hongoh Y, Miyagishima SY, Hattori M, Piel J, Fukatsu T. 2013. Defensive bacteriome symbiont with a drastically reduced genome. Curr Biol 23:1478–1484.

10. Carvalho R, Reid R, Viswanathan N, Gramajo H, Julien B. 2005. The biosynthetic genes for disorazoles, potent cytotoxic compounds that disrupt microtubule formation. Gene 359:91–98.

11. Lim S-K, Ju J, Zazopoulos E, Jiang H, Seo J-W, Chen Y, Feng Z, Rajski SR, Farnet CM, Shen B. 2009. Iso-migrastatin, migrastatin, and dorrigocin production in *Streptomyces platensis* NRRL 18993 is governed by a single biosynthetic machinery featuring an acyltransferase-less type I polyketide synthase. J Biol Chem 284:29746–56.

12. Dehn R, Katsuyama Y, Weber A, Gerth K, Jansen R, Steinmetz H, Höfle G, Müller R, Kirschning A. 2011. Molecular basis of elansolid biosynthesis: Evidence for an unprecedented quinone methide initiated intramolecular diels-alder cycloaddition/ macrolactonization. Angew Chemie - Int Ed 50:3882–3887.

13. Zhang F, He HY, Tang MC, Tang YM, Zhou Q, Tang GL. 2011. Cloning and elucidation of the FR901464 gene cluster revealing a complex acyltransferase-less polyketide synthase using glycerate as starter units. J Am Chem Soc 133:2452–2462.

14. Xie Y, Wang B, Liu J, Zhou J, Ma J, Huang H, Ju J. 2012. Identification of the biosynthetic gene cluster and regulatory cascade for the synergistic antibacterial antibiotics griseoviridin and viridogrisein in *Streptomyces griseoviridis*. ChemBioChem 13:2745–2757.

15. Mattheus W, Gao LJ, Herdewijn P, Landuyt B, Verhaegen J, Masschelein J, Volckaert G, Lavigne R. 2010. Isolation and purification of a new kalimantacin/batumin-related polyketide antibiotic and elucidation of its biosynthesis gene cluster. Chem Biol 17:149–159.

16. Weber T, Laiple KJ, Pross EK, Textor A, Grond S, Welzel K, Pelzer S, Vente A, Wohlleben W. 2008. Molecular analysis of the kirromycin biosynthetic gene cluster revealed β-alanine as precursor of the pyridone moiety. Chem Biol 15:175–188.

17. Seo JW, Ma M, Kwong T, Ju J, Lim SK, Jiang H, Lohman JR, Yang C, Cleveland J, Zazopoulos E, Farnet CM, Shen B. 2014. Comparative characterization of the lactimidomycin and iso-migrastatin biosynthetic machineries revealing unusual features for acyltransferase-less type I polyketide synthases and providing an opportunity to engineer new analogues. Biochemistry 53:7854–7865.

18. Cheng Y-Q, Tang G-L, Shen B. 2002. Identification and localization of the gene cluster encoding biosynthesis of the antitumor macrolactam leinamycin in *Streptomyces atroolivaceus* S-140 184:7013–7024.

19. Schneider K, Chen XH, Vater J, Franke P, Nicholson G, Borriss R, Süssmuth RD. 2007. Macrolactin is the polyketide biosynthesis product of the pks2 cluster of *Bacillus amyloliquefaciens* FZB42. J Nat Prod 70:1417–1423.

20. Biggins JB, Ternei MA, Brady SF. 2012. Malleilactone, a polyketide synthase-derived virulence factor encoded by the cryptic secondary metabolome of *Burkholderia pseudomallei* group pathogens. J Am Chem Soc 134:13192–13195.

21. El-Sayed AK, Hothersall J, Cooper SM, Stephens E, Simpson TJ, Thomas CM. 2003. Characterization of the mupirocin biosynthesis gene cluster from *Pseudomonas fluorescens*. NCIMB 10586. Chem Biol 10:419–430.

22. Sucipto H, Wenzel SC, Müller R. 2013. Exploring chemical diversity of α-pyrone antibiotics: Molecular basis of myxopyronin biosynthesis. ChemBioChem 14:1581–1589.

23. Simunovic V, Zapp J, Rachid S, Krug D, Meiser P, Müller R. 2006. Myxovirescin A biosynthesis is directed by hybrid polyketide synthases/nonribosomal peptide synthetase, 3-hydroxy-3-methylglutaryl-CoA synthases, and trans-acting acyltransferases. ChemBioChem 7:1206–1220.

24. Kampa A, Gagunashvili AN, Gulder TAM, Morinaka BI, Daolio C, Godejohann M, Miao VPW, Piel J, Andrésson ÓS. 2013. Metagenomic natural product discovery in lichen provides evidence for a family of biosynthetic pathways in diverse symbioses. Proc Natl Acad Sci U S A 110:E3129-37.

25. Matilla MA, Stöckmann H, Leeper FJ, Salmond GPC. 2012. Bacterial biosynthetic gene clusters encoding the anti-cancer haterumalide class of molecules: Biogenesis of the broad spectrum antifungal and anti-oomycete compound, oocydinA. J Biol Chem 287:39125-39138.

26. Zhao C, Ju J, Christenson SD, Smith WC, Song D, Zhou X, Shen B, Deng Z. 2006. Utilization of the methoxymalonyl-acyl carrier protein biosynthesis locus for cloning the oxazolomycin biosynthetic gene cluster from *Streptomyces albus* JA3453. J Bacteriol 188:4142–4147.

27. Kwan JC, Donia MS, Han AW, Hirose E, Haygood MG, Schmidt EW. 2012. Genome streamlining and chemical defense in a coral reef symbiosis. Proc Natl Acad Sci U S A 109:20655–20660.

28. Piel J. 2002. A polyketide synthase-peptide synthetase gene cluster from an uncultured bacterial symbiont of *Paederus beetles*. Proc Natl Acad Sci U S A 99:14002–14007.

29. Bertin MJ, Vulpanovici A, Monroe EA, Korobeynikov A, Sherman DH, Gerwick L, Gerwick WH. 2016. The phormidolide biosynthetic gene cluster: a *trans*-AT PKS pathway encoding a toxic macrocyclic polyketide. ChemBioChem 17:164–173.

30. Mast Y, Weber T, Gölz M, Ort-Winklbauer R, Gondran A, Wohlleben W, Schinko E. 2011. Characterization of the "pristinamycin supercluster" of *Streptomyces pristinaespiralis*. Microb Biotechnol 4:192–206.

31. Fisch KM, Gurgui C, Heycke N, van der Sar SA, Anderson SA, Webb VL, Taudien S, Platzer M, Rubio BK, Robinson SJ, Crews P, Piel J. 2009. Polyketide assembly lines of uncultivated sponge symbionts from structure-based gene targeting. Nat Chem Biol 5:494–501.

32. Pistorius D, Müller R. 2012. Discovery of the rhizopodin biosynthetic gene cluster in *Stigmatella aurantiaca* sg a15 by genome mining. ChemBioChem 13:416–426.

33. Zou Y, Yin H, Kong D, Deng Z, Lin S. 2013. A *trans*-acting ketoreductase in biosynthesis of a symmetric polyketide dimer SIA7248. ChemBioChem 14:679–683.

34. Irschik H, Kopp M, Weissman KJ, Buntin K, Piel J, Müller R. 2010. Analysis of the sorangicin gene cluster reinforces the utility of a combined phylogenetic/retrobiosynthetic analysis for deciphering natural product assembly by *trans*-AT PKS. ChemBioChem 11:1840–1849.

35. Elshahawi SI, Trindade-Silva AE, Hanora A, Han AW, Flores MS, Vizzoni V, Schrago CG, Soares CA, Concepcion GP, Distel DL, Schmidt EW, Haygood MG. 2013. Boronated tartrolon antibiotic produced by symbiotic cellulose-degrading bacteria in shipworm gills. Proc Natl Acad Sci U S A 110:E295-304.

36. Nguyen T, Ishida K, Jenke-Kodama H, Dittmann E, Gurgui C, Hochmuth T, Taudien S, Platzer M, Hertweck C, Piel J. 2008. Exploiting the mosaic structure of *trans*-acyltransferase polyketide synthases for natural product discovery and pathway dissection. Nat Biotechnol 26:225–233.

37. Liu X, Biswas S, Berg MG, Antapli CM, Xie F, Wang Q, Tang MC, Tang GL, Zhang L, Dreyfuss G, Cheng YQ. 2013. Genomics-guided discovery of thailanstatins A, B, and C as pre-mRNA splicing inhibitors and antiproliferative agents from *Burkholderia thailandensis* MSMB43. J Nat Prod 76:685–693.

38. Fukuda D, Haines AS, Song Z, Murphy AC, Hothersall J, Stephens ER, Gurney R, Cox RJ, Crosby J, Willis CL, Simpson TJ, Thomas CM. 2011. A natural plasmid uniquely encodes two biosynthetic pathways creating a potent anti-MRSA antibiotic. PLoS One 6:1–9.

39. Pancrace C, Barny M-A, Ueoka R, Calteau A, Scalvenzi T, Pédron J, Barbe V, Piel J,

Humbert J-F, Gugger M. 2017. Insights into the *Planktothrix* genus: Genomic and metabolic comparison of benthic and planktic strains. Sci Rep 7:41181.

40. Pulsawat N, Kitani S, Nihira T. 2007. Characterization of biosynthetic gene cluster for the production of virginiamycin M, a streptogramin type A antibiotic, in *Streptomyces virginiae*. Gene 393:31–42.

41. Mahenthiralingam E, Song L, Sass A, White J, Wilmot C, Marchbank A, Boaisha O, Paine J, Knight D, Challis GL. 2011. Enacyloxins are products of an unusual hybrid modular polyketide synthase encoded by a cryptic *Burkholderia ambifaria* genomic island. Chem Biol 18:665–677.
